# Role of succinyl substituents in the mannose-capping of lipoarabinomannan and control of inflammation in *Mycobacterium tuberculosis* infection

**DOI:** 10.1371/journal.ppat.1011636

**Published:** 2023-09-05

**Authors:** Zuzana Palčeková, Andrés Obregón-Henao, Kavita De, Amanda Walz, Ha Lam, Jamie Philp, Shiva Kumar Angala, Johnathan Patterson, Camron Pearce, Sophie Zuberogoitia, Charlotte Avanzi, Jérôme Nigou, Michael McNeil, Juan F. Muñoz Gutiérrez, Martine Gilleron, William H. Wheat, Mercedes Gonzalez-Juarrero, Mary Jackson

**Affiliations:** 1 Mycobacteria Research Laboratories, Department of Microbiology, Immunology and Pathology, Colorado State University, Fort Collins, Colorado, United States of America; 2 Institut de Pharmacologie et de Biologie Structurale, IPBS, Université de Toulouse, CNRS, UPS, Toulouse, France; 3 Department of Microbiology, Immunology and Pathology, Colorado State University, Fort Collins, Colorado, United States of America; University of Massachusetts Medical School, UNITED STATES

## Abstract

The covalent modification of bacterial (lipo)polysaccharides with discrete substituents may impact their biosynthesis, export and/or biological activity. Whether mycobacteria use a similar strategy to control the biogenesis of its cell envelope polysaccharides and modulate their interaction with the host during infection is unknown despite the report of a number of tailoring substituents modifying the structure of these glycans. Here, we show that discrete succinyl substituents strategically positioned on *Mycobacterium tuberculosis* (*Mtb*) lipoarabinomannan govern the mannose-capping of this lipoglycan and, thus, much of the biological activity of the entire molecule. We further show that the absence of succinyl substituents on the two main cell envelope glycans of *Mtb*, arabinogalactan and lipoarabinomannan, leads to a significant increase of pro-inflammatory cytokines and chemokines in infected murine and human macrophages. Collectively, our results validate polysaccharide succinylation as a critical mechanism by which *Mtb* controls inflammation.

## Author summary

*Mycobacterium tuberculosis*, the agent of tuberculosis (TB) in humans, remains the leading cause of death due to a single infectious agent. In the context of the increasing incidence of multidrug-resistant TB infections, understanding the key physiological processes allowing this paramount pathogen to adapt and thrive in the stressful environment of the host may prove useful in designing innovative therapeutic strategies.

The covalent modifications of cell envelope (lipo)polysaccharides with discrete substitutions such as (amino)sugars, amino acids, phosphates or acyl groups is a well-established strategy used by a variety of bacteria to enhance virulence and resistance to antibiotics and host defense mechanisms. While increasing evidence points to *M*. *tuberculosis* similarly fine-tuning the structure of its glycans with various tailoring substituents, their biological significance remains essentially unknown. We here show that the succinylation of the dominant cell envelope polysaccharides of *M*. *tuberculosis* governs their decoration with additional, biologically active, glycosyl substituents and promotes the establishment of infection by controlling inflammation.

## Introduction

The covalent modification of bacterial (lipo)polysaccharides with various (amino)sugars, amino acids, phosphates or acyl groups has been reported to control their biosynthesis and export in addition to impacting the interactions of bacteria with the host and their resistance to antimicrobials [1–7]. While devoid of the canonical lipopolysaccharides (LPS) and (lipo)teichoic acids found in other prokaryotes, mycobacteria produce two distinct heteropolysaccharides, arabinogalactan (AG) and lipoarabinomannan (LAM). Both polymers are critical to the integrity of the cells and are known to be modified by discrete covalent modifications [8]. The biological significance of these modifications remains largely unknown.

**Fig 1:**
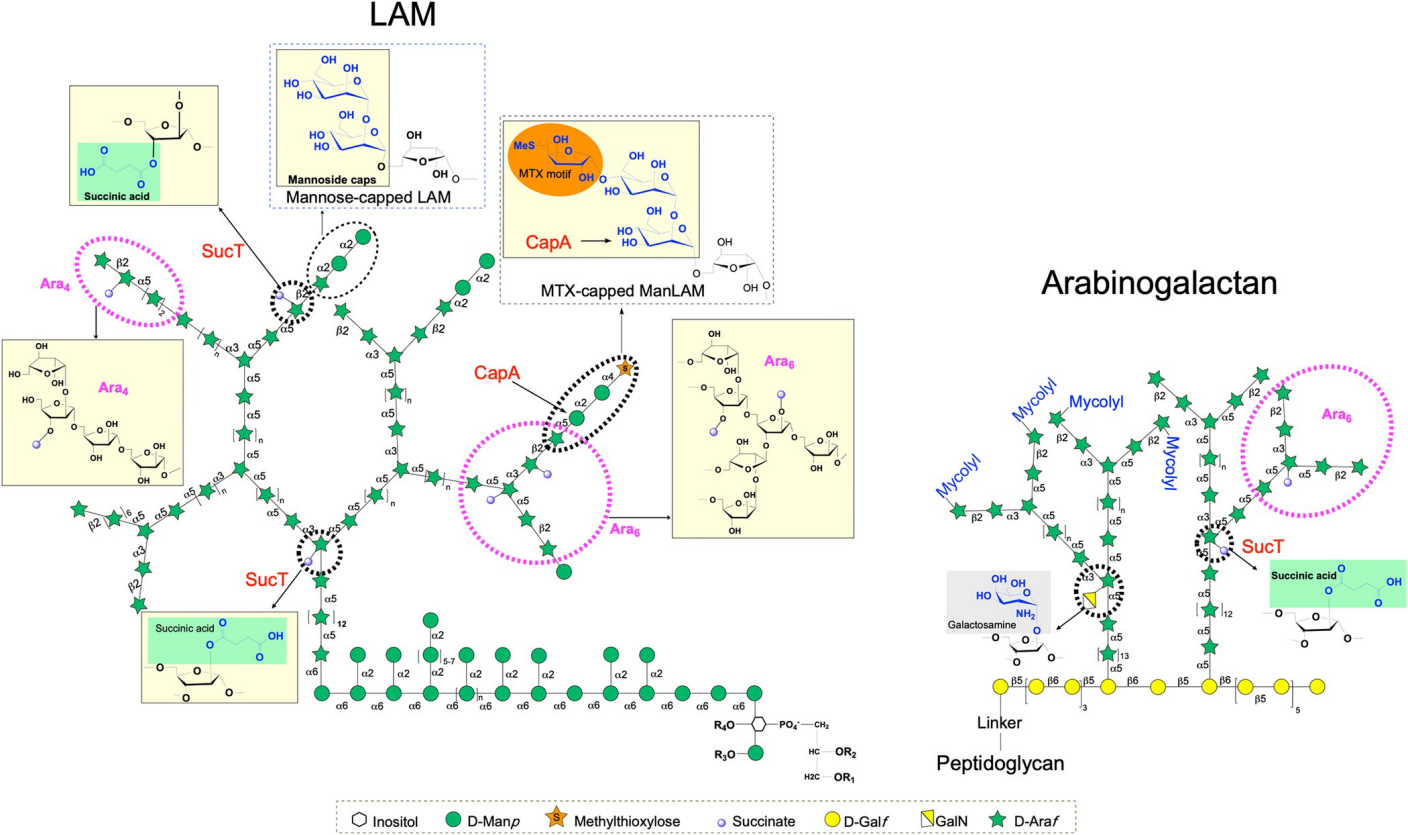
Detail of the covalent substituents modifying the arabinan domains of AG and LAM in *Mtb*. The various chemical modifications found on AG, LM, and LAM are shown in colored boxes: Mannoside caps: yellow boxes; succinyl substituents: green; methylthioxylose (MTX): orange, galactosamine: gray. The succinylation of the mycolylated chains in AG has been reported to be diminished or absent compared to that of non-mycolylated chains [22]. Ara_5_ motifs, not shown on this figure, are thought to be extended linear Ara_4_ motifs harboring one additional Ara*f* residue [10].

LAM has three distinct structural domains including a phosphatidylinositol anchor, a mannan core which consists of α(1→6)-linked mannosyl residues substituted at some positions with single mannoses or mannan side chains, and an arabinan consisting of α(1→5)-linked arabinosyl residues with some α(1→3)-branching [Fig 1]. Three distinct motifs, known as Ara_4_, Ara_5_ and Ara_6_, make up the non-reducing arabinan termini of LAM, all ending in β(1→2)-linked arabinosyl units [9,10] [Fig 1]. *M*. *tuberculosis* (*Mtb*) AG is made of a galactan domain and one or two arabinan chains that are very similar in structure to that of LAM except for the fact its non-reducing arabinan termini exclusively consist of Ara_6_ motifs [11,12] [Fig 1]. Decorating these relatively conserved LAM and AG core structures are a number of discrete, precisely positioned, covalent substituents. The non-reducing arabinan termini of LAM, in particular, display considerable species-specific structural heterogeneity which is known to be key to the biological activity of the entire molecule. One of the best characterized arabinan termini substituents are mannoside caps found on the LAM of *Mtb* and a number of other pathogenic slow-growing mycobacteria. These substituents have been associated with numerous biological activities aimed at promoting intracellular survival and immune evasion. Studies conducted for the most part using purified LAM have indeed implicated the mannoside caps of LAM in the ability of the lipoglycan to bind to C-type lectins (mannose receptor, dectin-2 and DC-SIGN), inhibit phagosomal maturation and *Mtb*-induced apoptosis and autophagy, impair signaling pathways of phagocytic cells and T-lymphocytes, and stimulate an anti-inflammatory response [9,13–16]. The role of other covalent modifications of LAM and AG in mycobacterial physiology and infection is less well defined [8].

We and others reported on the presence of succinyl substituents on the arabinan domains of AG and LAM of *Mtb*, *M*. *smegmatis*, *M*. *abscessus* and *M*. *bovis* BCG, and on the identity of the succinyltransferase (named SucT) responsible for their addition to both polysaccharides [17–19] [Fig 1]. An interesting feature shared by *sucT*-deficient mutants in all *Mycobacterium* spp. analyzed to date relates to their altered cell surface properties as evidenced by changes in their colony morphology, biofilm forming capacity, surface hydrophobicity and/or aggregative properties [17–18,20–21]. On the basis of these observations, we proposed that the succinylation of the two major polysaccharides of the mycobacterial cell envelope served to modulate, probably through indirect charge-mediated effects, the surface properties of bacilli thereby allowing the bacterium to adjust the way it interacts with its environment [18]. The fact that the prevalence of succinyl substituents on *Mtb* LAM increases during murine infection supports a role in host adaptation [10]. That succinylation may also control the elongation, branching and other modifications of LAM and AG has been proposed but never conclusively answered [22]. Structural analyses of the AG and LAM produced by *sucT* mutants of the fast-growing *M*. *smegmatis* and *M*. *abscessus* revealed that they display wild-type structures [17–18]. In contrast, and although not known to be impacted in the succinylation of AG and LAM at the time, a *sucT* mutant of the slow-growing *Mycobacterium* species, *M*. *marinum*, was reported to produce a LAM devoid of mannoside caps, with a higher degree of branching of both its mannan and arabinan domains, and a reduced acylation of its phosphatidylinositol anchor [20].

To further explore the physiological implications of LAM and AG succinylation in mycobacteria and address potential differences between slow- and fast-growing species, we here studied the impact of disrupting *sucT* on the biosynthesis of AG and LAM in the major human pathogen, *Mtb*, and interactions of this bacterium with innate immune cells.

## Results

### Impact of sucT disruption on the structure of Mtb LAM

A *Mtb* CDC1551 mutant, *Mtb sucT*::*Tn*, harboring a transposon insertion at position 614 of the *MT1616* gene was obtained from BEI Resources [23]. MT1616 is 100, 75, 61 and 83% identical at the amino acid level with SucT from *Mtb* H37Rv (Rv1565c), *M*. *smegmatis*, *M*. *abscessus*, and *M*. *marinum*, respectively. Complemented mutant strains were generated by transforming *Mtb sucT*::*Tn* with either the replicative plasmid pMVGH1-*Rv1565c* harboring a wild-type (WT) copy of the *Mtb sucT* gene under control of the *hsp60* promoter (*Mtb sucT*::*Tn* comp) or the integrative plasmid pMV306H harboring a WT copy of *Mtb sucT* similarly expressed under control of the *hsp60* promoter (*Mtb sucT*::*Tn* comp-int).

**Fig 2:**
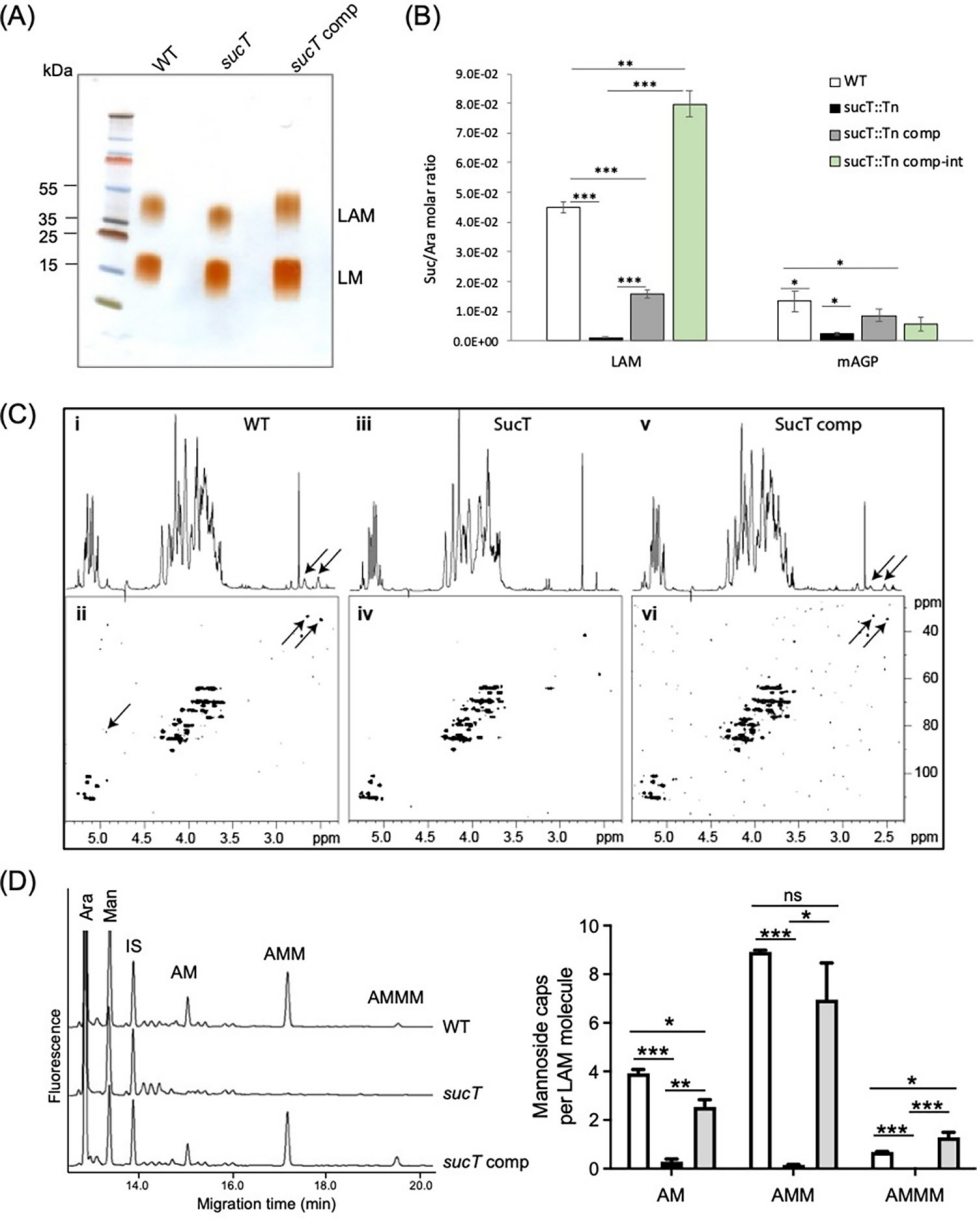
Biochemical analysis of LAM and AG prepared from WT *Mtb* CDC1551, the *sucT* mutant and the complemented *sucT* mutant strains. (A) Electrophoretic mobility of LM and LAM. Lipoglycans purified from WT *Mtb* CDC1551, *Mtb sucT*::*Tn*, and *Mtb sucT*::*Tn*/pMVGH1-*Rv1565c* (*Mtb sucT*::*Tn* comp) were run on a 10–20% Tricine gel followed by periodic acid-silver staining. The results presented are representative of two independent SDS-PAGE runs using different lipoglycan preparations from each strain. (B) Succinate content of AG and LAM. GC/MS-based quantification of succinates and arabinose residues in the same LAM and mAGP samples prepared from the WT, mutant and complemented mutant strains. An assumption is made here based on other structural analyses (alditol acetate and glycosyl linkage analyses presented in S1 through S4 Tables) that the arabinan domains of LAM and AG are not significantly altered in the mutant. Results are expressed as average ± SD succinate/arabinose molar ratios from three technical replicates. Asterisks denote statistical differences (*p < 0.05; **p < 0.005; ***p < 0.0005, Student’s *t*-test). The two complemented mutant strains shown here are: *Mtb sucT*::*Tn*/pMVGH1-*Rv1565c* (*sucT* expressed under control of the *hsp60* promoter from a replicative plasmid; *Mtb sucT*::*Tn* comp) and *Mtb sucT*::*Tn*/pNIP40b-*Rv1565c* (*sucT* expressed under control of the *hsp60* promoter from an integrative plasmid; *Mtb sucT*::*Tn* comp-int). (C) NMR analysis of LAM. Shown are the 1D ^1^H (i, iii and v) and 2D ^1^H-^13^C (ii, iv and vi) HMQC NMR spectra of LAM purified from *Mtb* CDC1551 WT, *Mtb sucT*::*Tn* and *Mtb sucT*::*Tn* comp. Arrows point to the signals typifying succinates. The 1D ^1^H spectra of the WT and complemented mutant LAM show the characteristic two pseudo-triplets (J = 6.5 Hz) of similar intensity at 2.50 and 2.65 ppm assigned to methylene groups of succinyl units (panels i and v). Their corresponding carbons were characterized at 34.7 and 33.3 ppm, respectively, on the 2D ^1^H-^13^C HMQC spectra (panels ii and vi). These signals are absent from the *sucT* mutant 1D ^1^H and 2D ^1^H-^13^C HMQC NMR spectra (panels iii and iv). (D) Quantification of mannooligosaccharide caps in the LAM from WT *Mtb*, the *sucT* mutant and the complemented mutant strain, *Mtb sucT*::*Tn* comp. Left panel: Capillary electrophoresis cap profile of LAM prepared from the three strains. Right panel: Abundance of the different cap motifs per LAM molecule. White bars, WT *Mtb* CDC1551; black bars, *Mtb sucT*::*Tn*; grey bars, *Mtb sucT*::*Tn* comp. IS, internal standard, mannoheptose-APTS; AM, Man*p*-(α1→5)-Ara-APTS (mono-mannoside cap); AMM, Man*p*-(α1→2)-Man*p*-(α1→5)-Ara-APTS (di-mannoside cap); AMMM, Man*p*-(α1→2)-Man*p*-(α1→2)-Man*p*-(α1→5)-Ara-APTS (tri-mannoside cap). The results are shown are averages and standard deviations from three technical replicates. Asterisks denote statistical differences pursuant to the Student’s *t*-test (***p<0.0001; **p<0.005; *p<0.05. ns = not significant).

Analysis of the lipoglycans from the WT, mutant and complemented mutant strains by SDS-PAGE pointed to the slightly faster migration of the LAM from *Mtb sucT*::*Tn* relative to the WT and complemented mutant LAM [Fig 2A], which was opposite to what had been reported for the LAM produced by *sucT* mutants of *M*. *smegmatis* and *M*. *abscessus* [17–18] and was suggestive of a smaller size LAM in the case of *Mtb sucT*::*Tn*.

The degree of succinylation of *Mtb sucT*::*Tn* LAM relative to that produced by the WT and complemented mutant strains was first quantified by gas chromatography-mass spectrometry (GC/MS) analysis of succinates (as their dibutyl ester derivatives) obtained from purified LAM from each strain [17]. The results showed an almost complete absence of succinates in the mutant LAM [Fig 2B]. This result was confirmed by liquid chromatography-mass spectrometry (LC/MS) analysis of the oligoarabinosides released by the WT, mutant and complemented mutant LAM upon digestion with the *Cellulomonas gelida* endoarabinanase [Table 1 and S1 Fig], and 1D ^1^H and 2D ^1^H-^13^C HMQC NMR analyses of the LAM from the same strains [Fig 2C].

**Table 1:** LC/MS analysis of the unmodified and covalently modified oligoarabinosides released from the LAM of *Mtb* CDC1551 WT, the *sucT* mutant and the complemented *sucT* mutant upon *Cellulomonas gelida* endoarabinanase digestion. Shown in bold letters are the relative percentages of total (including, unmodified and covalently modified) Ara_4_, Ara_5_ and Ara_6_ oligoarabinosides released upon *Cellulomonas gelida* endoarabinanase digestion of LAM from the different strains and the individual representation (expressed as percentages) of unmodified and covalently modified oligoarabinosides within each group. Suc, succinate; MTX, methylthioxylose; Man_x_, mannoside cap containing x mannose residues. This experiment was performed once on the same LAM preparations from the WT, mutant (“*sucT*”) and complemented mutant (“*sucT* comp”) as used in the GC/MS, NMR and capillary electrophoresis analyses shown in Fig 2.

	WT	*sucT*	*sucT* comp		WT	*sucT*	*sucT* comp		WT	*sucT*	*sucT* comp
%Ara_4_/(Ara_4_+Ara_5_+Ara_6_)	43.7	44.8	46.4	%Ara_5_/(Ara_4_+Ara_5_+Ara_6_)	20.0	13.5	16.9	%Ara_6_/(Ara_4_+Ara_5_+Ara_6_)	36.3	41.7	36.7
Ara_4_	13.4	99.0	48.0	Ara_5_	19.7	91.3	33.5	Ara_6_	13.0	97.6	28.2
Man_1_Ara_4_	6.2	-	2.5	Man_1_Ara_5_	2.0	-	0.4	Man_1_Ara_6_	2.6	0.5	1.6
Man_2_Ara_4_	48.8	0.6	34.1	Man_2_Ara_5_	3.1	4.8	6.7	Man_2_Ara_6_	3.1	0.5	4.2
MTX1Man_1_Ara_4_	-	-	-	MTX1Man_1_Ara_5_	-	-	-	MTX1Man_1_Ara_6_	-	-	-
Man_3_Ara_4_	0.9	-	0.2	Man_3_Ara_5_	2.7	-	2.6	Man_3_Ara_6_	6.6	0.9	4.1
MTX1Man_2_Ara_4_	2.4	0.4	4.3	MTX1Man_2_Ara_5_	-	0.6	1.2	MTX1Man_2_Ara_6_	-	0.4	1.4
Man_4_Ara_4_	-	-	-	Man_4_Ara_5_	20.4	-	21.1	Man_4_Ara_6_	51.6	-	31.8
MTX1Man_3_Ara_4_	-	-	0.3	MTX1Man_3_Ara_5_	-	-	-	MTX1Man_3_Ara_6_	-	-	-
Man_5_Ara_4_	-	-	-	Man_5_Ara_5_	3.2	-	6.9	Man_5_Ara_6_	7.8	-	13.7
MTX1Man_4_Ara_4_	-	-	-	MTX1Man_4_Ara_5_	1.6	-	4.2	MTX1Man_4_Ara_6_	4.8	-	7.5
Man_6_Ara_4_	-	-	-	Man_6_Ara_5_	1.9	3.3	2.2	Man_6_Ara_6_	0.6	0.1	1.9
MTX1Man_5_Ara_4_	-	-	-	MTX1Man_5_Ara_5_	-	-	1.4	MTX1Man_5_Ara_6_	0.8	-	3.2
Ara_4_+suc	25.0	-	9.3	Ara_5_+suc	38.1	-	16.7	Ara_6_+suc	7.0	-	2.3
Man_1_Ara_4_+suc	0.9	-	-	Man_1_Ara_5_+suc	4.9	-	0.5	Man_1_Ara_6_+suc	-	-	-
Man_2_Ara_4_+suc	1.6	-	0.5	Man_2_Ara_5_+suc	2.2	-	1.2	Man_2_Ara_6_+suc	0.4	-	0.1
MTX1Man_1_Ara_4_+suc	-	-	-	MTX1Man_1_Ara_5_+suc	-	-	-	MTX1Man_1_Ara_6_+suc	-	-	-
Man_3_Ara_4_+suc	-	-	-	Man_3_Ara_5_+suc	0.2	-	0.8	Man_3_Ara_6_+suc	-	-	-
MTX1Man_2_Ara_4_+suc	-	-	-	MTX1Man_2_Ara_5_+suc	-	-	-	MTX1Man_2_Ara_6_+suc	-	-	-
Man_4_Ara_4_+suc	-	-	-	Man_4_Ara_5_+suc	-	-	-	Man_4_Ara_6_+suc	1.7	-	-
MTX1Man_3_Ara_4_+suc	-	-	-	MTX1Man_3_Ara_5_+suc	-	-	-	MTX1Man_3_Ara_6_+suc	-	-	-
Man_5_Ara_4_+suc	0.8	-	0.8	Man_5_Ara_5_+suc	-	-	-	Man_5_Ara_6_+suc	-	-	-
MTX1Man_4_Ara_4_+suc	-	-	-	MTX1Man_4_Ara_5_+suc	-	-	-	MTX1Man_4_Ara_6_+suc	-	-	-
Man_6_Ara_4_+suc	-	-	-	Man_6_Ara_5_+suc	-	-	0.6	Man_6_Ara_6_+suc	-	-	-
MTX1Man_5_Ara_4_+suc	-	-	-	MTX1Man_5_Ara_5_+suc	-	-	-	MTX1Man_5_Ara_6_+suc	-	-	-

Another striking observation that arose from the LC/MS analysis of the oligoarabinosides released by the different LAMs upon digestion with the endoarabinanase was the dramatically reduced presence of mannoside caps (and thus, concomitantly, methylthio-D-xylose [MTX] motif) on the non-reducing arabinan termini of the mutant LAM (98.4, 79.4 and 97% reduction on the Ara_4_, Ara_5_ and Ara_6_ termini, respectively, compared to the WT strain) [Figs 1 and S1 and Table 1]. This result was confirmed by mild acid hydrolysis of purified LAM followed by analysis of liberated oligosaccharides by capillary electrophoresis which revealed a 96.7% decrease in the amount of combined mono-, di-, and tri-mannoside caps per molecule of LAM in the *sucT* mutant relative to the WT strain [Fig 2D]. Mannoside capping was restored in the complemented mutant [Table 1 and Figs 2D and S1]. The absence of mannoside caps on the mutant LAM likely explains its faster migration on SDS-PAGE since purified LAM from mutants deficient in the expression of the mannosyltransferase required for the addition of the first Man*p* residue of the caps, CapA, was reported earlier to display a similar migration pattern [24–26].

Quantitative analyses of the alditol acetate and per-*O*-methylated alditol acetate derivatives of the WT, mutant and complemented mutant LM revealed near identical structures in all three strains [S1 and S2 Tables]. Parallel analyses conducted on their LAM, in contrast, showed an increase in the Ara*f* to Man*p* ratio of the mutant LAM that accompanied an increase in terminal Ara*f* and a decrease in terminal Man*p* and 2-linked Man*p* residues, consistent with the loss of mannoside caps [S1 and S2 Tables]. The relative proportion of Ara_4_, Ara_5_ and Ara_6_ arabinan termini of LAM was otherwise comparable in all three strains as was the relative abundance of β-2-linked Ara*f* residues which serve as acceptors for the mannoside caps of LAM [Tables 1 and S2]. The fatty acyl composition of the LM and LAM lipid anchors of all three strains was also comparable, except for a slight increase in the tuberculostearic acid content of LM in the mutant and complemented mutant strains at the expense of oleic acid [S3 Table].

Since a *sucT* mutant of *M*. *marinum* was reported earlier to display a reduction in the acylation of polar forms of phosphatidylinositol mannosides (PIM) [20], which are metabolic precursors of LM and LAM, we further analyzed the PIM composition of the WT, mutant and complemented strains by thin-layer chromatography and LC/MS, but did not observe any decrease in the phosphatidylinositol hexamannoside content of the mutant relative to the other two strains or changes in the acylation pattern of these glycolipids [S2 Fig].

In conclusion, the inactivation of *sucT* in *Mtb* led to the abolition of succinylation and almost complete loss of mannoside caps and MTX motifs on LAM. Loss of mannoside capping was the only phenotype shared by the *Mtb* and *M*. *marinum sucT* mutants since *Mtb sucT*::*Tn* produced otherwise a WT-like LAM with regard to its mannan and arabinan domains and phosphatidylinositol anchor.

### The Mtb sucT mutant produces an AG devoid of succinyl substituents

The degree of succinylation of AG in *Mtb sucT*::*Tn* relative to the WT and complemented strains was determined by submitting purified mycolyl-AG-peptidoglycan (mAGP) complex to the same butanolysis procedure as for LAM [17–18]. This analysis revealed a dramatic decrease in the succinate content of the mutant mAGP which was partially or totally restored in the complemented mutants [Fig 2B]. The residual succinates in *Mtb sucT*::*Tn* mAGP are tentatively attributed to the activity of another succinyltransferase capable of succinylating the arabinan domain of AG or as yet unknown other positions of AG, peptidoglycan or mycolic acids. Analyses of the monosaccharide composition [S4 Table] and glycosyl linkages [S5 Table] of the WT, mutant and complemented mutant AG otherwise revealed near identical AG structures in all three strains. The degree of mycolylation of the mutant AG was also comparable to that of the WT and complemented mutant strains [S4 Table].

### The succinyl substituents of LAM are required for the mannose capping of LAM by the decaprenyl phosphomannose-dependent mannosyltransferase, CapA

To probe the reasons underlying the loss of LAM mannoside caps in *Mtb sucT*::*Tn*, we next sought to determine whether succinylation of LAM governed the ability to the decaprenyl-phosphomannose-dependent mannosyltransferase CapA to mannosylate the non-reducing arabinan termini of LAM [25] [Fig 1]. This hypothesis was tested genetically by expressing *Mtb capA* in the backgrounds of *M*. *abscessus* WT and an *M*. *abscessus* mutant deficient in SucT activity. Like other rapidly growing mycobacteria, *M*. *abscessus* is naturally deficient in CapA activity, consistent with the fact that it produces a LAM devoid of any form of capping [18]. We reasoned that if succinylation of the non-reducing arabinan termini of LAM was required for CapA to add the first Man*p* residue of the cap in *Mtb*, then this enzyme should be active in *M*. *abscessus* WT which produces succinylated arabinan termini, but not in a *M*. *abscessus sucT* knock-out whose corresponding termini are devoid of succinyl substituents [18]. Both the WT and *sucT* mutant strains of *M*. *abscessus* were reported previously to display comparable relative abundances of β-2-linked Ara*f* residues which serve as acceptors for the mannoside caps of LAM [18]. *M*. *abscessus* WT and Δ*sucT* were transformed with the *capA* expression plasmid pVV16-MT1671 [25,27]. The oligoarabinosides released by LAM purified from the *capA*-expressing WT and Δ*sucT* strains upon digestion with the *Cellulomonas gelida* endoarabinanase were next analyzed by LC/MS. The results which are presented in [Table 2] clearly pointed to the addition of mannoside caps containing up to three mannose residues onto the Ara_4_, Ara_5_ and Ara_6_ non-reducing arabinan termini of *M*. *abscessus* LAM in the WT strain expressing *capA* which were not detected in WT *M*. *abscessus* lacking the *capA* expression plasmid. Mannose-capping also occurred in the *M*. *abscessus sucT* mutant expressing *capA*, albeit at a much-reduced level compared to the *capA*-expressing WT strain. Indeed, mannosylated Ara_4_ and Ara_6_ oligoarabinosides in the *capA*-expressing mutant only occurred at 3% and 4.8% the levels observed in the *capA*-expressing WT strain, respectively, and no mannosylated Ara_5_ oligoarabinosides were formed in the mutant background. Thus, consistent with the finding of limited mannoside capping in the *Mtb sucT*::*Tn* mutant, the absence of LAM succinylation did not completely abolish the mannosylation of the non-reducing arabinan termini in *M*. *abscessus* Δ*sucT* expressing *capA* but drastically reduced the efficiency of CapA-mediated mannosyl transfer.

**Table 2:** LC/MS analysis of the mannose capping of LAM in *capA*-expressing *M*. *abscessus* strains either proficient or deficient in LAM succinylation. Shown in bold letters are the relative percentages of total (including, unmodified and covalently modified) Ara_4_, Ara_5_ and Ara_6_ oligoarabinosides released upon *Cellulomonas gelida* endoarabinanase digestion of LAM from *M*. *abscessus* WT and Δ*sucT* expressing or not *capA* (from pVV16-*MT1671*) and the individual representation (expressed as percentages) of unmodified and covalently modified oligoarabinosides within each group. Suc, succinate; Man_x_, mannoside cap containing x mannose residues.

	WT	WT +*capA*	Δ*sucT*	Δ*sucT +capA*
**%Ara**_**4**_ **/(Ara**_**4**_ **+Ara**_**5**_ **+Ara**_**6**_ **)**	**45.9**	**44.6**	**36.5**	**37.5**
Ara_4_	85.1	48.2	100	99.2
Man_1_Ara_4_	-	20.1	-	0.3
Man_2_Ara_4_	-	0.1	-	0.4
Man_3_Ara_4_	-	0.8	-	0.1
Man_4_Ara_4_	-	0.3	-	-
Ara_4_+suc	14.9	25.1	-	-
Man_1_Ara_4_+suc	-	5.4	-	-
Man_2_Ara_4_+suc	-	-	-	-
Man_3_Ara_4_+suc	-	-	-	-
Man_4_Ara_4_+suc	-	-	-	-
**%Ara**_**5**_ **/(Ara**_**4**_ **+Ara**_**5**_ **+Ara**_**6**_ **)**	**17.4**	**21.2**	**14.0**	**13.0**
Ara_5_	63.4	28.3	100	100
Man_1_Ara_5_	-	6.6	-	-
Man_2_Ara_5_	-	-	-	-
Man_3_Ara_5_	-	-	-	-
Man_4_Ara_5_	-	-	-	-
Ara_5_+suc	36.6	53.2	-	-
Man_1_Ara_5_+suc	-	11.9	-	-
Man_2_Ara_5_+suc	-	-	-	-
Man_3_Ara_5_+suc	-	-	-	-
Man_4_Ara_5_+suc	-	-	-	-
**%Ara**_**6**_ **/(Ara**_**4**_ **+Ara**_**5**_ **+Ara**_**6**_ **)**	**36.7**	**34.2**	**49.5**	**49.5**
Ara_6_	88.9	47.5	99.9	98.3
Man_1_Ara_6_	-	22.9	-	1.2
Man_2_Ara_6_	-	11.1	-	0.1
Man_3_Ara_6_	-	0.5	-	0.4
Man_4_Ara_6_	-	-	-	-
Ara_6_+suc	11.1	17.6	0.1	-
Man_1_Ara_6_+suc	-	-	-	-
Man_2_Ara_6_+suc	-	0.4	-	-
Man_3_Ara_6_+suc	-	-	-	-
Man_4_Ara_6_+suc	-	-	-	-

### Impact of AG and LAM succinylation on the growth, surface properties, and drug susceptibility of Mtb

The study by independent groups of *sucT*-deficient mutants of *M*. *avium*, *M*. *smegmatis*, *M*. *marinum* and *M*. *abscessus* has led to the conclusion that the loss of AG and LAM succinylation alters, in a species-specific manner, one or more of the following phenotypes reflective of the surface properties of the bacilli: colony morphology, surface hydrophobicity, biofilm-forming capacity, and propensity to aggregate in liquid broth [17–18,20–21]. While the *sucT* mutant of *Mtb* failed to show any obvious changes in colony morphology, acid-fast staining [S3A Fig] or surface hydrophobicity [S3B Fig], and did not hyper-aggregate in liquid broth, it grew at a slower rate than WT *Mtb* CDC1551 in laboratory medium [S3C Fig]. Complementation of the *Mtb sucT* mutant with a WT copy of the *sucT* gene expressed either from a replicative plasmid under control of the constitutive *hsp60* promoter (*Mtb sucT*::*Tn* comp) or from an integrative plasmid under control of the same promoter (*Mtb sucT*::*Tn* comp-int), while working to restore the succinylation of LAM and AG [Fig 2], failed to restore WT growth [S3C Fig]. Although frameshift mutations in the *glpK* and *ponA2* genes of the mutant and complemented mutant were identified by whole genome sequencing [S6 Table], it is unlikely that they contribute to the pronounced growth retardation of these strains in 7H9-ADC-Tween 80 medium (devoid of glycerol supplement) given that neither *glpK* or *ponA2* null mutants display such growth defects under these conditions [28–30]. In the absence of additional secondary mutations in *Mtb sucT*::*Tn* comp-int relative to *Mtb sucT*::*Tn* that might have explained the lack of restoration of growth of the complemented mutant [S6 Table], we presently have no explanation for this phenotype other than suspecting improper or improperly regulated levels of expression of *sucT* in the complemented strain preventing WT growth in 7H9-ADC-Tween 80. Alternatively, the presence of a C-terminal hexahistidine tag on the SucT protein produced by the complemented strain may hamper its activity or ability to productively interact with other proteins. Of note, we experienced similar difficulties in trying to restore the axenic growth of *M*. *smegmatis* and *M*. *abscessus sucT* KO mutants upon genetic complementation (including with WT *sucT* orthologs devoid of His_6_-tag) [17–18]. Interestingly, a recent Tn-Seq study found the *sucT* gene to become critical to *M*. *smegmatis* growth when asymmetric cell elongation was impaired (i.e., upon disruption of the mycobacterial divisome factor, *lamA*) [31]. While no clear explanation was provided for this observation, this result points to an important impact either of cell envelope glycan succinylation or the presence of the SucT protein itself (e.g., as part of a multiprotein complex) during cell elongation and division and thus replicative growth.

Consistent with a previous report [32], drug susceptibility testing revealed a modest but reproducible 2-to 4-fold increase in the susceptibility of *Mtb* CDC1551 *sucT* mutant to imipenem and carbenicillin relative to the WT and complemented mutant strains [S7 Table]. Susceptibility to the two β-lactams reverted towards WT levels in the complemented mutant.

### Immune responses induced by Mtb sucT::Tn in murine bone marrow-derived macrophages

**Fig 3:**
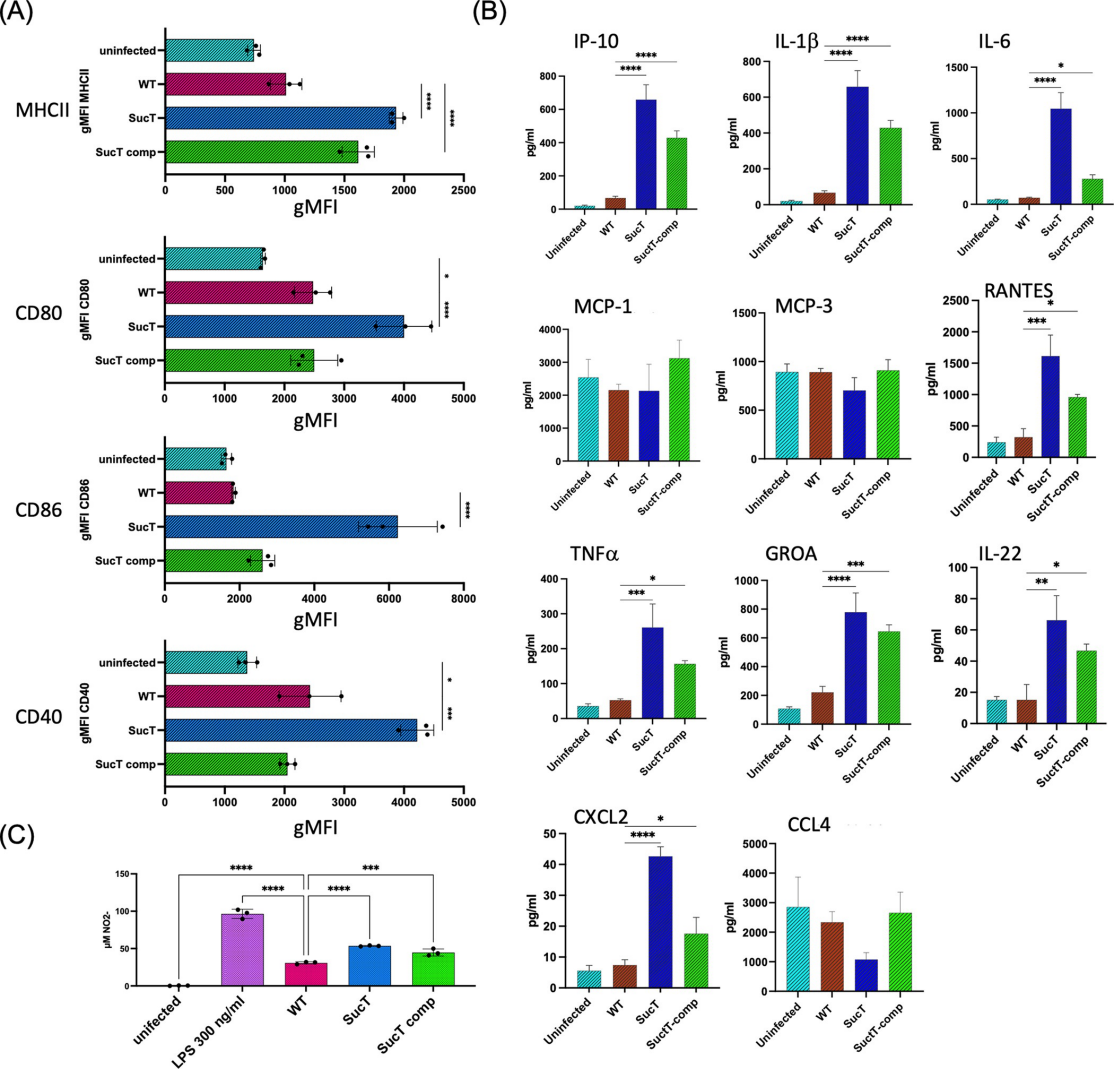
Evaluation of immune activation and chemokine/cytokine secretion by C3HeB/FeJ BMMΦ infected with *Mtb* CDC1551 WT, the *sucT* mutant and the complemented mutant strain. C3HeB/FeJ BMMΦ were infected with either WT *Mtb* CDC1551, *Mtb sucT*::*Tn* (“SucT”) or *Mtb sucT*::*Tn* comp (“SucT comp”) and allowed to adhere for 2 h. Cells were washed to remove extracellular bacteria and subsequently harvested 48 h post-infection for activation marker, chemokine/cytokine and NO release analysis. (A) Levels of expression of activation markers for MHCII, CD80, CD86 and CD40 was determined by flow cytometry. (B) Culture supernatants were analyzed for chemokine/cytokine secretion by multiplex immunoassay using Luminex. (C) NO production was determined using the Griess Reagent test. Immune activation, chemokine/cytokine release and NO data for triplicate samples were analyzed using an ordinary one-way ANOVA with *p≤0.05, **p≤0.01, ***p≤0.005 and ****p≤0.001. Error bars represent the mean spanning the SD of each replicate. Experiment was repeated two times.

We reported earlier on the reduced uptake and intracellular replication and survival of a *M*. *abscessus sucT* mutant compared to the WT parent and complemented mutant strains in macrophages and epithelial cells [18]. To investigate potential effects of AG and LAM succinylation on the interactions of *Mtb* with innate immune cells, mouse bone marrow-derived macrophages (BMMΦ) from C3HeB/FeJ mice were infected with WT *Mtb* CDC1551, the *sucT* mutant and the complemented mutant, and the different strains compared for their intracellular survival and ability to induce expression of innate immunity activation markers and chemokine/cytokine secretion. Accordingly, macrophages were infected with the three strains at an MOI of 5. Similar numbers of bacilli were recovered from infected macrophages 2 h after infection indicative of equivalent numbers of infecting *Mtb* strains [S4A Fig]. Determination of intracellular CFUs 5 days post-infection showed that the intracellular persistence of the *sucT* mutant was slightly but significantly less than that measured for the WT and complemented mutant strains [S4A Fig]. Interestingly, determination of the level of expression of macrophage antigen presentation/processing and activation markers (MHCII, CD80, CD86 and CD40) by flow cytometry after 48 h of infection [Fig 3A] revealed that all markers were significantly increased in cells infected with the *sucT* mutant. Cells infected with the *Mtb sucT*::*Tn* comp mutant, in contrast, demonstrated expression levels that were not significantly different from their WT counterparts [Fig 3A]. Analysis of chemokines revealed that, in comparison to infection with WT *Mtb* or *Mtb sucT*::*Tn* comp, secretion of IP-10, CXCL2, GROA (GRO-alpha) and RANTES were significantly increased with the *sucT* mutant, as was the secretion of key pro-inflammatory cytokines (TNFα, IL-6, IL1-β and IL-22), indicative of macrophages poised for increased proinflammatory state [Fig 3B]. Infection with the *sucT*::*Tn* mutant, when compared to *Mtb* WT, indicated no significant differences in expression of MCP-1, MCP-3 or CCL4. Finally, culture supernatants from cells infected with the *sucT* mutant presented significantly more nitric oxide (NO). This release of NO was comparable to levels observed in BMMΦ treated with LPS [Fig 3C].

### Innate immune responses induced by Mtb sucT::Tn in human primary macrophages

**Fig 4:**
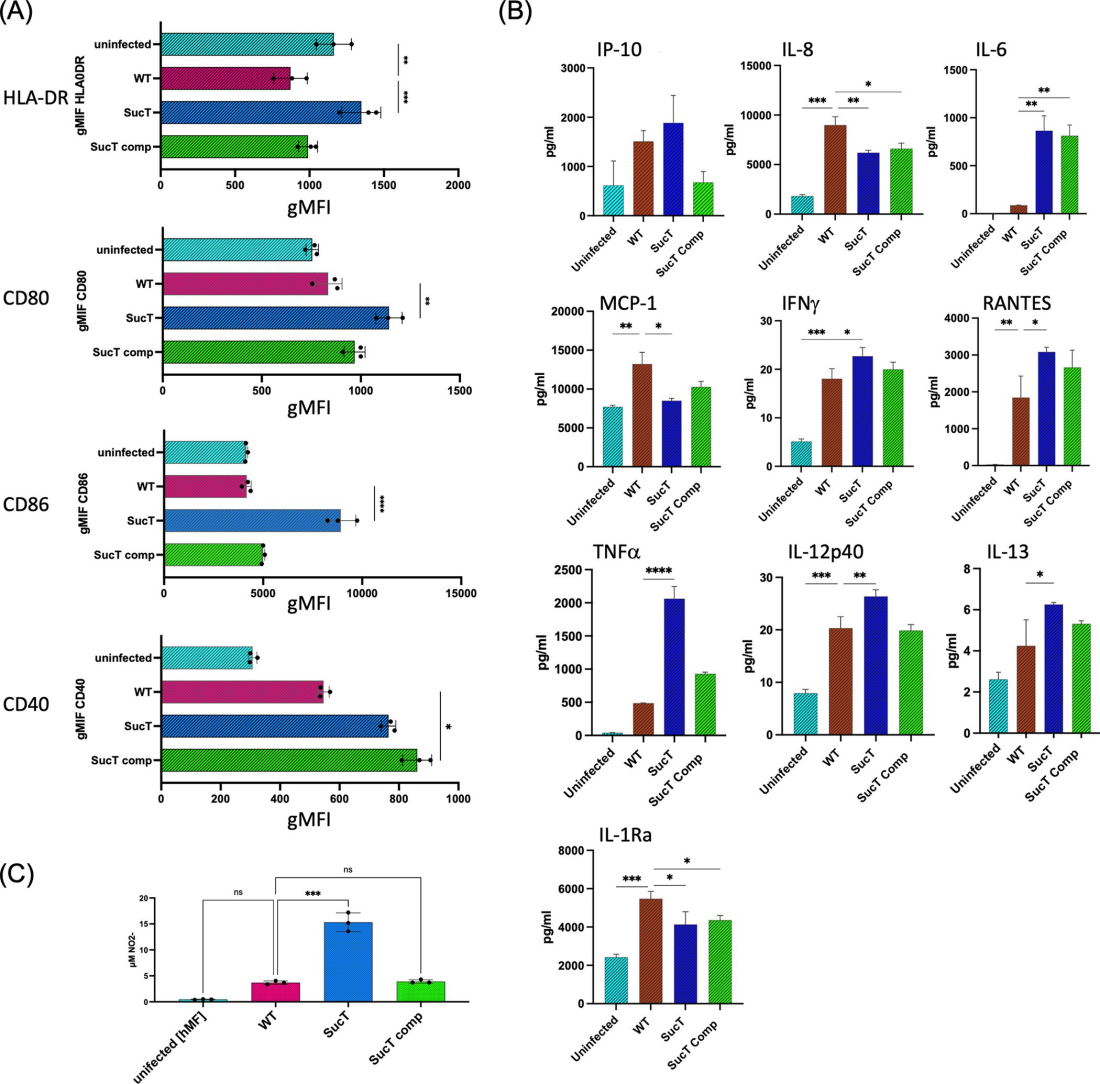
Evaluation of immune activation and chemokine/cytokine secretion by M-CSF-differentiated MDM infected with *Mtb* CDC1551 WT, the *sucT* mutant and the complemented mutant strain. Human monocyte derived macrophages (MDM) were infected with either WT *Mtb* CDC1551, *Mtb sucT*::*Tn* (“SucT”) or *Mtb sucT*::*Tn* comp (“SucT comp”) for 2 h. Cells were then washed to remove extracellular bacteria and were subsequently harvested 48 h post-infection for activation marker, chemokine/cytokine and NO release analysis. (A) Levels of expression of activation markers for HLA-DR, CD80, CD86 and CD40 was determined by flow cytometry. (B) Cytokines and chemokines in culture supernatants were analyzed by multiplex immunoassay using Luminex. (C) NO secretion was determined by the Griess Reagent kit. Immune activation, chemokine/cytokine release and NO data for triplicate samples were analyzed using an ordinary one-way ANOVA with *p≤0.05, **p≤0.01, ***p≤0.005 and ****p≤0.001. Error bars represent the mean spanning the SD of each replicate. Experiment was repeated two times.

Similar trends as far as immune responses and intracellular survival were observed in infected human macrophages *in vitro* as was observed in murine BMMΦ [Figs 4 and S4B]. Compared to WT and/or *Mtb sucT*::*Tn* comp-infected cells, *sucT* mutant-infected monocyte-derived macrophages (MDM) differentiated from PBMC obtained from whole blood of healthy human donors consistently displayed significant increases in human antigen presentation/processing activation markers (HLA-DR, CD80, CD86 and CD40) [Fig 4A]. With the exception of CD40, expression levels of the same markers in cells infected with the complemented mutant were not significantly different from those measured in WT-infected cells. Interestingly, despite an overall lower CFU recovery from *Mtb sucT*::*Tn*-infected cells when compared to infection with WT *Mtb* (but comparable bacterial load to the complemented mutant) [S4B Fig], there was a significant increase in expression of the chemokine RANTES along with reductions of MCP-1. Pro-inflammatory cytokines (IL-6, IFNγ, TNFα, and IL-12p40) as well as the anti-inflammatory cytokine IL-13 increased, whereas release of IL-1Ra (an IL-1β inhibitor) was reduced by infection with the mutant strain [Fig 4B]. Genetic complementation largely resulted in release of chemokines and cytokines that were not significantly different from their cognate WT infection with the noted exceptions of IL-8, IL-6 and IL-1Ra that did not demonstrate expression levels comparable to WT [Fig 4B]. Finally, NO release in culture supernatants from MDM infected with the *sucT* mutant was significantly more pronounced compared to cells infected with WT *Mtb* or the complemented mutant strain [Fig 4C]. Overall, human macrophages released considerably less NO than was observed in the mouse BMMΦ, consistent with earlier observations.

Collectively, results from human MDM are again consistent with the notion that strategically placed succinyl substituents in major cell envelope polysaccharides have an immunomodulatory function that promotes the establishment of *Mtb* infection by limiting or altering host pro-inflammatory and immune regulatory responses.

## Discussion

The molecular processes leading to the assembly of AG, PIM, LM and LAM in the cell envelope are expected to be tightly regulated [9,33–37]. Whether minor covalent modifications of these glycans might act as molecular signals controlling their biosynthesis and export, as was shown with other prokaryotic polysaccharides, had not yet been established. In Gram-negative bacteria, the acylation, phosphorylation and L-Ara_4_N modification of lipid A are critical to the proper biosynthesis and/or export of this biosynthetic precursor to the periplasmic side of the plasma membrane, and the translocation of LPS to the outer membrane [7,38]. Likewise, modifications of the nonreducing terminal residues of O-antigen polysaccharides and S-layer glycans by methyl and phosphate groups is a well-established strategy used by a number of Gram-positive and Gram-negative microorganisms to control the chain length and export of polysaccharides assembled and exported through ABC-transporter-dependent pathways [39]. Precedents for the existence of similar regulatory processes in mycobacteria are scarce. The transient acetylation of the mycolic acid moiety of trehalose monomycolate by the essential acetyltransferase TmaT appears to be a prerequisite for the export of trehalose monomycolate to the periplasmic space of Corynebacteria and Mycobacteria [40–41]. Preliminary studies on the biosynthesis of mycobacterial cytoplasmic polysaccharides known as the methylglucose lipopolysaccharides suggest that the acylation of these glycans controls both the position at which the methylation of glucosyl residues occurs [42] and the fatty acid-binding properties of the end products [43]. The results of our *capA* expression studies in *sucT*-proficient and *sucT*-deficient *M*. *abscessus* strains represent the first example of a discrete covalent modification controlling, most likely through charge-mediated effects, one of the key aspects of the biosynthesis of *Mtb* LAM: The addition of terminal mannoside caps by the decaprenyl-phosphomannose-dependent mannosyltransferase, CapA.

Another critical role of SucT-mediated polysaccharide succinylation revealed by our macrophage infection studies is the impact this tailoring modification has on the interactions of *Mtb* with innate immune cells. The hyper-inflammatory phenotype of the *sucT* mutant in murine and human macrophages suggests that the succinylation of AG and/or LAM in *Mtb* has an anti-inflammatory function delaying the activation of immune responses and possibly promoting or ensuring survival of the bacilli.

Early studies aimed at determining the role of LAM in mycobacterial virulence showed that *Mtb* LAM is able to alter a number of macrophage and dendritic cell functions associated with protective immunity. The mannoside caps of LAM, in particular, were shown to have profound implications on the biological activities of the entire molecule and were thus proposed to play an important role in virulence and the control of inflammation [9,13–16]. Of note, these caps may themselves be substituted with an α(1→4)-linked methylthio-D-xylose (MTX) residue [44] [Fig 1] whose immunomodulatory and anti-oxidative properties have been proposed to further enhance bacterial survival during infection [45–47]. The majority of the biological properties of mannoside-capped LAM, however, were inferred from cell-free studies using purified LAM (as summarized in ref. [15]). It is not until the discovery of *capA* and the construction of capless *capA* mutants that it actually became possible to measure the contribution of mannoside-capped LAM to pathogenesis in the context of live bacteria. The outcome of these whole cell-based studies came as a surprise to the field when disrupting *capA* failed to show any clear effect on the survival and replication of *M*. *marinum*, *M*. *bovis* BCG or *Mtb* in macrophage and animal models of infection (zebrafish, C57BL/6 and BALB/c mice) [24,26,48]. Likewise, an analysis of the immune responses (TNF-α, IL-17, IFNγ, NO) induced by an *Mtb capA*-deficient mutant compared to the WT parent strain in mouse bone-marrow-derived macrophages and the lungs and spleens of infected BALB/c mice failed to reveal any significant differences between the two strains [48]. It was proposed that other mannosylated ligands at the cell surface of whole bacilli (e.g., polar forms of phosphatidylinositol mannosides, glycoproteins and other as yet unidentified cell envelope constituents that share with mannosylated LAM the same α(1→2)-linked oligomannoside appendage) or other modulators of phagosome-lysosome fusion may play a more important role or, at least, have the ability to compensate for the loss of LAM mannoside-capping in the interactions of mycobacteria with host cells [24,48]. An important outcome of the present study is the realization that the control of LAM mannose-capping in *Mtb* may in fact only occur in the context of the addition or removal of succinates to the arabinan domain as opposed to solely being controlled at the level of CapA expression or activity. If that is the case, the effect of losing mannoside caps on LAM may only become apparent (i.e., detectable by immune cells) when AG and LAM succinylation is concurrently decreased or lost, both covalent substituents acting synergistically to achieve a particular outcome during infection. The results of infection experiments with the *sucT* mutant, however, should be interpreted cautiously given the broader direct and indirect effects succinyl substituents may have on the overall charge of AG and LAM and, thus, cell surface and cell envelope properties of mycobacterial bacilli [17–18,20]. Indeed, it is possible that the hyperinflammatory phenotype of *Mtb sucT*::*Tn* results from more complex and multifactorial cell envelope-related changes than the only loss of mannoside and MTX caps on LAM. To begin to address the mechanisms underlying the hyperinflammatory phenotype of this mutant, future investigations will aim to compare the virulence and immunopathogenicity of *sucT*, *capA* and MTX mutants of *Mtb* in murine models of TB infection and determining whether succinylation affects the way mannose- and MTX-capped LAM and other immunologically active surface ligands are presented to immune cells.

## Materials and methods

### Bacterial strains and growth conditions

*Mtb* CDC1551 and *M*. *abscessus* ATCC 19977 were grown under agitation at 37°C in Middlebrook 7H9 medium supplemented with 10% albumin-dextrose-catalase (ADC) (BD Sciences) and 0.05% Tween 80, in minimal Sauton’s medium with 0.05% tyloxapol, or on Middlebrook 7H11 agar supplemented with 10% oleic acid-albumin-dextrose-catalase (OADC) (BD Sciences). Zeocin (100 μg mL^-1^), hygromycin (50 μg mL^-1^) and kanamycin (25 μg mL^-1^ for *Mtb*; 100 μg mL^-1^ for *M*. *abscessus*) were added to the culture media as needed. *Mtb* CDC1551 *Mtb sucT*::*Tn* was obtained from BEI Resources. For genetic complementation, the *Mtb sucT* gene PCR-amplified using primers Rv1565c.fw (5’- ccaattccatatgttgaccctgtcgccgcctc -3’) and Rv1565c.rv (5’- cccaagcttccaccagtcggtgttcgccgc– 3’) was cloned downstream of the *hsp60* promoter in the replicative plasmid, pMVGH1 [49], yielding pMVGH1-*Rv1565c*. Alternatively, a WT copy of *sucT* was stably expressed under control of the *hsp60* promoter from the integrative plasmid, pNIP40b [50]. To this end, the entire *sucT* gene, C-terminal His_6_ tag and *hsp60* promoter were cut out from pMVGH1-*Rv1565c* with *Xba*I and *Hpa*I restriction enzymes and cloned into the blunted *Xba*I site of pNIP40b. *M*. *abscessus* Δ*sucT* was reported earlier^18^. *Mtb* CDC1551 WT, *Mtb sucT*::*Tn* and *Mtb sucT*::*Tn* comp-int (mutant complemented with an integrative plasmid) were whole genome sequenced as described further in the text, and the results are presented in S6 Table.

### Preparation and analysis of lipids, lipoglycans and arabinogalactan

Extraction of total lipids, lipoglycans and mycolyl-AG-peptidoglycan (mAGP) complex from *Mtb* followed the same procedures as we used earlier in the characterization of the *M*. *smegmatis* and *M*. *abscessus sucT* mutants [17–18]. Lipoglycans were purified by gel exclusion chromatography and analyzed by SDS-PAGE on commercial Novex 10–20% Tricine gels stained with periodic acid Schiff reagent.

Structural analyses of PIMs, lipoglycans and mAGP followed earlier procedures [17–18,51]. Briefly, 1 mg of mAGP, 50 μg of LM and 50 μg of LAM were used for permethylation and alditol acetates preparation and analyzed by GC/MS to determine monosaccharide composition and glycosyl linkage patterns. Succinates were analyzed and quantified by GC/MS analysis as their butyl succinate derivatives obtained from either 20 μg of purified LAM or 1 mg of mAGP. The presence of capping motifs at the non-reducing arabinan termini of LAM from *Mtb* and *M*. *abscessus* expressing *capA* was analyzed by LC/MS after digestion of LAM with *Cellulomonas gelida* endoarabinanase. The endoarabinanase digestion products of LAM were directly analyzed by ultra-performance liquid chromatography (UPLC) on an Atlantis T3 column (Waters) using Waters Acquity UPLC H-Class system coupled to a Bruker MaXis Plus QTOF MS instrument according to the method described by De *et al*. [10]. The identity of the digested products was further confirmed by UPLC analysis after their overnight reduction with 10 mg mL^-1^ NaBD_4_ in 1 M NH_4_OH in ethanol followed by their per-*O*-methylation. The presence of mannoside capping motifs at the non-reducing arabinan termini of LAM from the various *Mtb* and *M*. *abscessus* strains was also analyzed by capillary electrophoresis as described previously [52].

The fatty acids esterifying the mannosylated phosphatidyl-*myo*-inositol anchor of LM and LAM were analyzed as their fatty acid methyl esters (FAME) by GC/MS. Briefly, 100 μg of LM and LAM were methanolyzed in 100 μL of 3M methanolic HCl by heating at 80°C overnight and extracted with *n*-hexane:water (1:1). FAMEs were analyzed on a Thermo Scientific TRACE 1310 Gas Chromatograph paired with a Thermo Scientific TSQ 8000 Evo Triple Quadrupole GC-MS/MS. Samples were run on a 30 m x 0.25 mm x 0.25 μm Zebron ZB-5HT Inferno capillary column (Phenomenex) at an initial temperature of 60°C. The temperature was increased to 375°C at a ramp rate of 20°C min^-1^ and held for 5 min. Data handling was carried out using the Thermo Scientific Chromeleon Chromatography Data System software.

Mycolic acids released from mAGP by treatment with 2 M trifluoroacetic acid were quantified by LC/MS as described earlier [22] on an Agilent 1260 Infinity chromatograph equipped with a 2.1 mm × 150 mm (3.5 μm particle size) XBridge reverse phase C18 column (Waters) coupled to an Agilent 6224 time-of-flight (TOF) mass spectrometer. Data were analyzed using the Agilent MassHunter software. The same instrument and LC/MS method were used to analyze the PIM profile from *Mtb* total lipid extracts.

NMR experiments were performed at 303K with a cryo-probed Bruker DRX600 spectrometer (Karlsruhe, Germany) using 2D ^1^H-^1^H TOCSY and ^1^H-^13^C HMQC sequences previously reported [53]. Native molecules were dissolved in D_2_O and analyzed in 200 x 5 mm 535-PP NMR tubes. Proton and carbon chemical shifts are expressed in ppm downfield from the signal of external acetone (δH 2.22 and δC 30.89).

### Acid-fast staining of Mtb bacilli

Bacteria were fixed with 1.5% formalin in 10% skim milk suspension and smeared on a microscope slide. The staining protocol followed the modified Kinyoun method and used TB Kinyoun Carbolfuchsin to stain the bacilli for 5 min, followed by decolorization with 3% HCl in ethanol, rinsing and counterstain with 0.3% methylene blue for 1 min.

### Hydrophobicity index

Relative hydrophobicities were assessed by the hexadecane partition procedure [54]. Briefly, 1 OD 650 nm unit PBS-washed cell suspension of each strain was mixed with 0.3 mL hexadecane by vortexing for 2 min. The hydrophobicity index is defined as the percentage reduction in the OD650 nm of the aqueous phase after partitioning with the hydrocarbon phase.

### Drug susceptibility testing

Minimum inhibitory concentration (MIC) values were determined in 7H9-OADC-tyloxapol in a total volume of 100 μL in 96-well microtiter plates. *Mtb* cultures grown to early log phase (OD600 nm ∼ 0.2) were diluted to OD600nm 0.01 and incubated in the presence of serial dilutions of the antibiotics for 10 days at 37°C. MICs were determined using the resazurin blue test [55].

### Ethics statement

The Institutional Animal Care and Use Committee of Colorado State University approved all animal studies. Studies were performed in accordance with recommendations of the Guide for the Care and Use of Laboratory Animals of the National Institutes of Health.

### Preparation of mouse bone marrow macrophages

Six to eight weeks old C3HeB/FeJ female mice were purchased from The Jackson Laboratory (Bar Harbor. ME). Bone marrow cells were harvested from tibias and femurs of C3HeB/FeJ mice. Bone marrow cells were flushed from bones by snipping the head of the bones and placing them up-side-down in sterile 0.5-mL Eppendorf tubes (with a hole in bottom) inserted into a larger 1.0 mL Eppendorf tube. The bones within Eppendorf tubes were centrifuged 15 seconds at 10,000 x *g* and the bone marrow cells were collected at the bottom of the enveloped Eppendorf tube. The bone marrow cells were differentiated into macrophages by incubation in complete RPMI medium (cRPMI supplemented with 10% heat-inactivated FBS, recombinant M-CSF or GM-CSF [Peprotech; 15–20 ng/mL], 0.1% of 55 mM 2-mercaptoethanol, 1% penicillin-streptomycin (Gibco, Gaithersburg, MD), 0.3% Sodium Bicarbonate (Gibco), 0.8% L-glutamine, 1% essential amino acids solution (Gibco) and 1% non-essential amino acids solution (Gibco)). Primary cells were cultured for 7 days in 24-well plates with media changes every 2 days. Adherent cells were selected as mature macrophages (BMMΦs) for further studies and used at passage 1.

### Procurement of human blood and preparation of MDM

Units of whole blood were obtained by under a material transfer agreement from the Garth England Blood Bank in Fort Collins. Whole blood was obtained from anonymous donors who have been deemed acceptable for blood donation through screening for Human Immunodeficiency Virus, and Human Hepatitis A, B and C. No blood was donated specifically for this work and was procured from the blood center as a quantity not sufficient (QNS) and suitable for clinical application and would otherwise be disposed. Hence, no Internal Review Board (IRB) requirement was necessary for the acquisition of these human blood specimens. Blood was centrifuged at 400 x *g* for 20 min and the top buffy coat was removed. The buffy coat was diluted 5-fold in sterile PBS and layered over a 10 mL pad of Ficoll-Paque PLUS (GE Healthcare Life Sciences, Pittsburgh, PA) and centrifuged again at 400 x *g* with the brake off. White blood cells (WBCs) were extracted from the interface, washed twice in Hanks Balanced Salt Solution (HBSS) and subsequently resuspended in DMEM containing 10% fetal bovine serum (FBS). PBMC were plated at 3.0–5.0×10^6^ cells/mL in 24-well plates (CellTreat, Pepperell, MA) and monocytes were allowed to adhere to plastic for 2–3 h at 37°C. Monocytes were washed, counted and 1 mL of 15% FBS, 2% autologous donor serum containing growth factors was added. In total, differentiation medium was DMEM (Gibco/Life Technologies, Carlsbad, CA) containing 15% FBS, 1% penicillin-streptomycin (Hyclone, Logan, UT), 0.01M Hepes buffer (Hyclone, Logan, UT), 1 mM sodium pyruvate (Hyclone, Logan, UT), 1% MEM non-essential amino acids solution (Hyclone, Logan, UT)) and 30 ng/mL recombinant human M-CSF (R & D Systems, Minneapolis, MS). Monocytes were allowed to differentiate for 5–7 days (as determined by flow cytometric analysis of HLA-DR and morphological changes upon microscopic inspection). The differentiation medium was replaced every 48–72 h depending on the condition of the cells and pH of the culture.

### Infection of human MDM and mouse bone marrow macrophages with Mtb

After 5–7 days of human monocyte differentiation to macrophages or mouse bone marrow differentiation to macrophages (BMMΦs), the DMEM was removed from the adherent cells and washed three times with pre-warmed PBS to remove the penicillin-streptomycin in the differentiation medium. All infection and harvesting procedures were performed in an approved BSL-3 facility. Log phase growth of *Mtb* strains were added to each well at an MOI of 5. Bacilli were resuspended in HBSS containing Mg^++^ and Ca^++^ and 2% autologous donor serum so that 250 μL of inoculum could be added to each well. Mouse BMMϕ were infected *Mtb* strains also at an MOI of 5 in 250 μl of complete DMEM without penicillin-streptomycin. The plates were briefly centrifuged for 5 min at 600 x *g* to facilitate bacillary interaction with either BMMΦ or MDM. In some cases, the cells were treated with 300 ng/mL LPS as a positive stimulatory control. At 2, 48 or 120 h, cells were washed thrice with PBS to remove the extracellular bacteria and either harvested for flow cytometry or lysed to count intracellular bacterial CFU by plating serial dilutions on 7H11-OADC agar plates.

### Flow cytometric analysis of infected human MDM and mouse bone marrow macrophages

Following infection, BMMΦ or MDM were removed from the plate using ice cold PBS containing 5 mM EDTA. Cells were then transferred to a 96-well round bottom plate washed and stained for 20 min with fluorochrome-conjugated anti-mouse or anti-human MHCII, CD80, CD86 or CD40. The plate was then centrifuged and the cells washed then resuspended in PBS containing 4% PFA for 15 min. The plate was then centrifuged as above and cells washed with FACS staining buffer (PBS containing 2% FBS and 2 mM sodium azide). Expression of activation markers was determined using a Gallios flow cytometer (Beckman-Coulter, Brea, CA). List mode data (LMD) file data was analyzed using Flowjo software (Treestar Inc., Ashland, OR).

### Multiplex immunoassay for cytokines and chemokines

Multiplex immunoassay using macrophage culture supernatants was performed using a Luminex bead-based multiplex ELISA kit (ProcartaPlex Mouse Cytokine & Chemokine Panel 1; 26plex, Invitrogen or MILLIPLEX Human Cytokine/Chemokine/Growth Factor Panel A, Millipore for MDM) according to the manufacturer’s instructions. Samples were fixed with 4% formaldehyde and subsequently washed prior to acquisition. Sample data were acquired on a MAGPIX instrument running xPONENT 4.3 software (Luminex Corp.) and analyzed using a five-parameter logistic model with an 80–120% standard acceptance range. Data was graphed using Graphpad Prism version 4.00.

### Measurement of NO_2_^-^ production

Nitric oxide release was measured from supernatants of macrophage cultures after 48 hours infected with the various *Mtb* strains or stimulated with LPS, using the Griess Reagent kit according to manufacturer’s protocol (Promega, Madison WI).

### Statistical analysis

Unless otherwise indicated in the figure legends, data are expressed as the mean ± SD values from triplicate assays. The statistical tests used in the different experiments are indicated in the corresponding figure legends. Calculations were performed using Graphpad Prism version 4.00 for Windows (San Diego California USA). P-values <0.05 were considered significant.

### Whole genome sequencing

Genomic DNA from *Mtb* CDC1551 WT, the *sucT* transposon mutant and the complemented mutant (*Mtb sucT*::*Tn* comp-int) was extracted from log-phase cultures grown in 7H9-ADC-Tween 80 using the Qiagen UCP Pathogen kit (Qiagen) following the manufacturer’s recommendations. Illumina libraries were prepared from 1 μg of mechanically fragmented DNA (300-bp, Covaris M instrument) using the Kapa HyperPrep kit as described previously [56] and quantified using the Qubit double-stranded DNA (dsDNA) BR assay kit (ThermoFisher Scientific). Fragments size was assessed on a Tape Station instrument (Agilent). Libraries were multiplexed and sequenced as 75-base-long single-end reads on an Illumina NextSeq 500 instrument. Reads were adapted and quality trimmed with Trimmomatic v0.33 [57] and mapped against the *Mtb* H37Rv reference genome (RefSeq NC_000962.3) and the plasmid used for complementation using Bowtie2 v2.2.5 [58]. Reads with mapping quality below 15 were filtered out, and duplicate reads were omitted from downstream analyses to avoid false SNP calls. SNP calling was done using VarScan v2.3.9 [59]. SNP calls were applied using the following cutoffs: minimum overall coverage of one non-duplicated read, minimum of five non-duplicated reads supporting the SNP, base quality score >15, and a SNP frequency above 80%. All VCF files were analyzed using SnpEff v5.0 [60] and the *Mtb* H37Rv annotation (RefSeq NC_000962.3). Vcf were merged using the component bcftools (option–merge) of the SAMtools package [61]. The sequencing data described in this publication have been submitted to the NCBI under BioProject # PRJNA991707.

## Supporting information

S1 Table Monosaccharidic composition of LM and LAM from WT *Mtb*, the *sucT* mutant and the complemented mutant strain. Reported values are averages ± standard deviations of three technical repeats and represent relative distribution in %. The complemented mutant strain (*Mtb sucT*::*Tn* comp) expresses WT *sucT* from pMVGH1-*Rv1565c*. Asterisks denote statistically significant differences between the WT and *sucT* mutant LM and LAM pursuant to the Student’s *t*-test (*P* < 0.05).
(PDF)

S2 Table Glycosyl linkage analysis of per-*O*-methylated LM and LAM. Reported values are averages ± standard deviations of three technical repeats and represent relative distribution in %. The complemented mutant strain (*Mtb sucT*::*Tn* comp) expresses WT *sucT* from pMVGH1-*Rv1565c*. Asterisks denote statistically significant differences between the WT and *sucT* mutant LM and LAM pursuant to the Student’s *t*-test (*P* < 0.05).
(PDF)

S3 Table Fatty acid composition of the mannosylated phosphatidyl-*myo*-inositol anchor of LM and LAM from WT *Mtb*, the *sucT* mutant and the complemented mutant strain. Reported values represent relative distribution in %. C19: tuberculostearic acid. The complemented mutant strain (*Mtb sucT*::*Tn* comp) expresses WT *sucT* from pMVGH1-*Rv1565c*.
(PDF)

S4 Table Monosaccharidic composition of mAGP from WT *Mtb*, the *sucT* mutant and the complemented mutant strain. Reported values are averages ± SD of three technical repeats and represent relative distribution in %. No statistically significant differences between strains were observed pursuant to the Student’s *t*-test (*P* > 0.05). The complemented mutant strain (*Mtb sucT*::*Tn* comp) expresses WT *sucT* from pMVGH1-*Rv1565c*.
(PDF)

S5 Table Glycosyl linkage analysis of per-*O*-methylated mAGP. Reported values are averages ± SD of three technical repeats and represent relative distribution in %. No statistically significant differences between strains were observed pursuant to the Student’s *t*-test (*P* > 0.05). The complemented mutant strain (*Mtb sucT*::*Tn* comp) expresses WT *sucT* from pMVGH1-*Rv1565c*.
(PDF)

S6 Table Whole genome sequencing of WT *Mtb* CDC1551, the *sucT* transposon mutant and the complemented mutant strain, *Mtb sucT*::*Tn* comp-int. (PDF)

S7 Table Susceptibility of the *Mtb sucT* mutant to antibiotics. MICs were determined in 7H9-OADC-tyloxapol using the resazurin blue test and MIC values are in μg mL^-1^. AMP, ampicillin; CRB, carbenicillin; CIP, ciprofloxacin; RIF, rifampicin; INH, isoniazid; GEN, gentamycin; EMB, ethambutol; STR, streptomycin; CAP, capreomycin; HYG, hygromycin; IMI, imipenem. MIC determinations were performed two to three times on independent culture batches. The *sucT* complemented mutant strain (*Mtb sucT*::*Tn* comp) is resistant to hygromycin due to the presence of complementation plasmid, pMVGH1-*Rv1565c*.
(PDF)

S1 Fig Negative ion liquid chromatography-mass spectrometry (LC-MS) analysis of the oligoarabinosides released by the WT, the *sucT* mutant and the complemented mutant LAM upon digestion with the *Cellulomonas gelida* endoarabinanase. Related to Table 1. Shown are extracted ion chromatograms (EICs) of the most abundant digestion products cleaved by *Cellulomonas gelida* endoarabinanase from the nonreducing end of LAM purified from the different strains. Several signals with identical exact masses for Ara_4_ (A) and Ara_6_ (D) oligosaccharides with *m/z* values of 545.1723 [M-H]^−^ and 809.2569 [M-H]^−^, respectively, reveal the possibility of more structural isomers of tetra- and hexa-arabinoside termini in *Mtb* LAM. Ions corresponding to Ara_4_+succinate at *m/z* 645.1887 [M-H]^−^ (B) and Ara_6_+succinate at *m/z* 909.2732 [M-H]^−^ (E) are missing in the *sucT* mutant but were detected in the LAM purified from the WT and complemented mutant strains. The most abundant mannoside-capped digestion products from *Mtb* WT LAM are Man_2_Ara_4_ presented as [M-H]^−^ ions at *m/z* 869.2780 (C), and Man_4_Ara_6_ presented as doubly charged [M-2H]^−2^ ions at *m/z* 728.2304 [M-2H]^−2^ (F). These ions were not detected in the *sucT* mutant but their presence was restored in the complemented mutant LAM. The complemented mutant strain used in this experiment (*Mtb sucT*::*Tn* comp) expresses WT *sucT* from pMVGH1-*Rv1565c*.
(PDF)

S2 Fig PIM composition of *Mtb* CDC1551 WT, the *sucT* mutant and the complemented mutant strain. (A) Thin-layer chromatography analysis of total lipids extracted from WT *Mtb* CDC1551, the *sucT* mutant and the complemented mutant strain. Total lipids were loaded on aluminum-backed silica gel 60-precoated plates F_254_ using chloroform:methanol:water (65:25:4; by vol.) as the eluent. TLCs were revealed by spraying with cupric sulfate (i) or α-naphthol (ii) and charring. The complemented mutant strain (*Mtb sucT*::*Tn* comp) expresses WT *sucT* from pMVGH1-*Rv1565c*. (B) LC/MS analysis of PIM. Shown is the relative distribution of PIMs in WT *Mtb* CDC1551 (WT), the *sucT* mutant and the complemented mutant strain in %. PIM is used to describe the global family of phosphatidylinositol mannosides that carries one to four fatty acids (attached to the glycerol, inositol and/or mannose) and one to six mannose residues. In Ac_X_PIM_Y_, x refers to the number of acyl groups esterified to available hydroxyls on the mannose or *myo*-inositol residues, y refers to the number of mannose residues; *e*.*g*. Ac_2_PIM_2_ corresponds to the phosphatidylinositol dimannoside PIM_2_ carrying two acyl groups attached to the glycerol (the diacylglycerol substituent), one acyl group esterified to the mannose residue and one acyl group esterified to the *myo*-inositol residue. The complemented mutant strain (*Mtb sucT*::*Tn* comp) expresses WT *sucT* from pMVGH1-*Rv1565c*.
(PDF)

S3 Fig Phenotypic characterization of *Mtb sucT*::*Tn*. (A) Acid-fast staining of *Mtb* CDC1551 WT, the *sucT* mutant (*sucT*::*Tn*) and the complemented mutant strain (*sucT*::*Tn* comp). (B) Surface hydrophobicity of *Mtb* CDC1551 WT and the *sucT* mutant (*sucT*::*Tn*). Relative hydrophobicities were assessed by the hexadecane partition procedure as described in the Materials and Methods. The hydrophobicity index (H) is defined as the percentage reduction in the OD650 nm of the aqueous phase after partitioning with the hydrocarbon phase. The reported values are averages ± SD of three technical repeats. No statistically significant differences were noted between strains (*P* > 0.05; Student’s *t*-test). (C) Growth kinetics of WT *Mtb* CDC1551, *Mtb sucT*::*Tn*, *Mtb sucT*::*Tn comp* and *Mtb sucT*::*Tn comp-int* in 7H9-ADC-Tween 80 (no glycerol) at 37°C.
(PDF)

S4 Fig Comparisons of *Mtb* CFU associated with C3HeB/FeJ BMMΦ and M-CSF-differentiated human MDM 2 hours and five days post-infection. C3HeB/FeJ BMMΦ (from Fig 3) (A) and rM-CSF-differentiated human monocyte derived macrophages (MDM from Fig 4) (B) were infected with either WT *Mtb* CDC1551, *Mtb sucT*::*Tn* or *Mtb sucT*::*Tn* comp and allowed to adhere for 2 h. Macrophages were subsequently lysed 2 h and 120 h post-infection and lysates were plated on 7H11-OADC agar plates for CFU counting. Shown are averages and standard deviations for triplicate wells. Data were analyzed using ordinary two-way ANOVA with * p≤0.01, **p≤0.005 and ****p≤0.0001; ns, not significant.
(PDF)

S1 Data Excel spreadsheet containing, in separate sheets, the underlying numerical data for Figs 2B, 2D, 3A, 3B, 3C, 4A, 4B, 4C, S3C, S4A and S4B. (XLSX)
