# Supplementary material for: Role of succinyl substituents in the mannose-capping of lipoarabinomannan and control of inflammation in Mycobacterium tuberculosis infection
Source: PLoS Pathog. 2023 Sep 5;19(9):e1011636. doi: 10.1371/journal.ppat.1011636 (PMC10503756; doi:10.1371/journal.ppat.1011636)
Supplement: S1 Table — Reported values are averages ± standard deviations of three technical repeats and represent relative distribution in %. The complemented mutant strain (Mtb sucT::Tn comp) expresses WT sucT from pMVGH1-Rv1565c. Asterisks denote statistically significant differences between the WT and sucT mutant LM and LAM pursuant to the Student’s t-test (P < 0.05). (PDF) [file ppat.1011636.s001.pdf]

<u>S1 Table</u>: Monosaccharidic composition of LM and LAM from WT *Mtb*, the *sucT* mutant and the complemented mutant strain. Reported values are averages ± standard deviations of three technical repeats and represent relative distribution in %. The complemented mutant strain (*Mtb sucT::Tn* comp) expresses WT *sucT* from pMVGH1-*Rv1565c*. Asterisks denote statistically significant differences between the WT and *sucT* mutant LM and LAM pursuant to the Student's *t*-test ($P < 0.05$).

(i) Monosaccharidic composition of LAM

	Ara*f*	Ino	Man*p*	Ara*f*/Man*p*
WT	60.8 ± 0.2	0.4 ± 0.1	38.8 ± 0.2	1.6 ± 0.0
sucT	74.7 ± 2.4*	0.2 ± 0.1*	25.1 ± 2.4*	3.0 ± 0.4
sucT comp	62.1 ± 0.6	0.4 ± 0.1	37.6 ± 0.7	1.7 ± 0.1

(ii) Monosaccharidic composition of LM[a]

	Ino	Man*p*
WT	0.8 ± 0.6	99.3 ± 0.6
sucT	0.8 ± 0.1	99.2 ± 0.1
sucT comp	1.1 ± 0.0	98.9 ± 0.0
