# Supplementary material for: Role of succinyl substituents in the mannose-capping of lipoarabinomannan and control of inflammation in Mycobacterium tuberculosis infection
Source: PLoS Pathog. 2023 Sep 5;19(9):e1011636. doi: 10.1371/journal.ppat.1011636 (PMC10503756; doi:10.1371/journal.ppat.1011636)
Supplement: S2 Table — Reported values are averages ± standard deviations of three technical repeats and represent relative distribution in %. The complemented mutant strain (Mtb sucT::Tn comp) expresses WT sucT from pMVGH1-Rv1565c. Asterisks denote statistically significant differences between the WT and sucT mutant LM and LAM pursuant to the Student’s t-test (P < 0.05). (PDF) [file ppat.1011636.s002.pdf]

<u>S2 Table</u>: Glycosyl linkage analysis of per-*O*-methylated LM and LAM.

Reported values are averages ± standard deviations of three technical repeats and represent relative distribution in %. The complemented mutant strain (*Mtb sucT::Tn* comp) expresses WT *sucT* from pMVGH1-*Rv1565c*. Asterisks denote statistically significant differences between the WT and *sucT* mutant LM and LAM pursuant to the Student's *t*-test ($P < 0.05$).

(i) Glycosyl linkage analysis of per-*O*-methylated LAM

	t-Ara*f*	2-Ara*f*	5-Ara*f*	3,5-Ara*f*	t-Man*p*	6-Man*p*	2,6-Man*p*	2-Man*p*	Ara*f*/Man*p*
WT	3.8 ± 0.4	7.1 ± 1.1	34.4 ± 1.6	11.3 ± 0.3	21.6 ± 1.2	8.3 ± 0.7	14.4 ± 0.3	0.5 ± 0.1	1.3 ± 0.0
sucT	11.5 ± 0.3*	10.7 ± 0.4*	32.4 ± 1.5	12.2 ± 0.2	13.1 ± 0.5*	6.5 ± 0.4*	13.7 ± 0.6	0.03 ± 0.0*	2.0 ± 0.1
sucT comp	4.4 ± 0.4	8.6 ± 0.7	35.7 ± 1.9	11.6 ± 0.6	21.3 ± 1.4	6.4 ± 0.5	13.3 ± 2.4	0.5 ± 0.2	1.5 ± 0.1

(ii) Glycosyl linkage analysis of per-*O*-methylated LM

	t-Man*p*	6-Man*p*	2,6-Man*p*
WT	43.5 ± 2.6	16.6 ± 0.6	39.9 ± 2.2
sucT	44.7 ± 1.7	19.4 ± 0.2*	35.9 ± 1.8
sucT comp	47.4 ± 1.6	16.5 ± 0.7	36.2 ± 1.1
