# Supplementary material for: Role of succinyl substituents in the mannose-capping of lipoarabinomannan and control of inflammation in Mycobacterium tuberculosis infection
Source: PLoS Pathog. 2023 Sep 5;19(9):e1011636. doi: 10.1371/journal.ppat.1011636 (PMC10503756; doi:10.1371/journal.ppat.1011636)
Supplement: S3 Table — Reported values represent relative distribution in %. C19: tuberculostearic acid. The complemented mutant strain (Mtb sucT::Tn comp) expresses WT sucT from pMVGH1-Rv1565c. (PDF) [file ppat.1011636.s003.pdf]

<u>S3 Table</u>: Fatty acid composition of the mannosylated phosphatidyl-*myo*-inositol anchor of LM and LAM from WT *Mtb*, the *sucT* mutant and the complemented mutant strain.

Reported values represent relative distribution in %. C19: tuberculostearic acid. The complemented mutant strain (*Mtb sucT::Tn* comp) expresses WT *sucT* from pMVGH1-*Rv1565c*.

	C16:0	C18:1	C18:0	C19
LM-WT	52.6	10.0	23.2	14.2
LM- *sucT*	52.8	0.7	25.9	20.6
LM- *sucT* comp	51.5	1.5	17.8	29.2
LAM- WT	49.8	1.0	28.0	21.2
LAM- *sucT*	51.7	0.0	26.4	22.0
LAM- *sucT* comp	52.1	0.0	22.0	25.9
