# Supplementary material for: Role of succinyl substituents in the mannose-capping of lipoarabinomannan and control of inflammation in Mycobacterium tuberculosis infection
Source: PLoS Pathog. 2023 Sep 5;19(9):e1011636. doi: 10.1371/journal.ppat.1011636 (PMC10503756; doi:10.1371/journal.ppat.1011636)
Supplement: S4 Table — Reported values are averages ± SD of three technical repeats and represent relative distribution in %. No statistically significant differences between strains were observed pursuant to the Student’s t-test (P > 0.05). The complemented mutant strain (Mtb sucT::Tn comp) expresses WT sucT from pMVGH1-Rv1565c. (PDF) [file ppat.1011636.s004.pdf]

<u>S4 Table</u>: Analysis of mAGP from WT *Mtb*, the *sucT* mutant and the complemented mutant strain.

(A) Monosaccharidic composition of mAGP.

Reported values are averages ± SD of three technical repeats and represent relative distribution in %. No statistically significant differences between strains were observed pursuant to the Student's *t*-test ($P > 0.05$). The complemented mutant strain (*Mtb sucT::Tn* comp) expresses WT *sucT* from pMVGH1-*Rv1565c*.

	Rha*p*	Ara*f*	Gal*f*	GlcNAc	GalNAc	MurNAc	Ara*f*/Gal*f*	mycolic acids/Rha*p*
WT	1.4 ± 0.2	42.1 ± 0.1	28.0 ± 1.8	16.7 ± 2.4	2.5 ± 0.5	9.5 ± 2.3	1.6 ± 0.0	5.2 ± 1.1
sucT	1.2 ± 0.2	42.2 ± 1.7	27.3 ± 0.2	16.1 ± 2.7	2.5 ± 0.3	10.7 ± 1.2	1.6 ± 0.1	5.7 ± 1.0
sucT comp	1.4 ± 0.1	41.0 ± 1.5	26.8 ± 1.0	15.8 ± 0.8	2.8 ± 0.3	12.2 ± 1.2	1.5 ± 0.1	5.0 ± 0.2
