# Supplementary material for: Role of succinyl substituents in the mannose-capping of lipoarabinomannan and control of inflammation in Mycobacterium tuberculosis infection
Source: PLoS Pathog. 2023 Sep 5;19(9):e1011636. doi: 10.1371/journal.ppat.1011636 (PMC10503756; doi:10.1371/journal.ppat.1011636)
Supplement: S5 Table — Reported values are averages ± SD of three technical repeats and represent relative distribution in %. No statistically significant differences between strains were observed pursuant to the Student’s t-test (P > 0.05). The complemented mutant strain (Mtb sucT::Tn comp) expresses WT sucT from pMVGH1-Rv1565c. (PDF) [file ppat.1011636.s005.pdf]

<u>S5 Table</u>: Glycosyl linkage analysis of per-*O*-methylated mAGP.

Reported values are averages ± SD of three technical repeats and represent relative distribution in %. No statistically significant differences between strains were observed pursuant to the Student's *t*-test ($P > 0.05$). The complemented mutant strain (*Mtb sucT::Tn* comp) expresses WT *sucT* from pMVGH1-*Rv1565c*.

	t-Ara*f*	2-Ara*f*	5-Ara*f*	3,5-Ara*f*	4-Rha*p*	t-Gal*f*	5-Gal*f*	6-Gal*f*	5,6-Gal*f*	Ara*f*/Gal*f*
WT	4.7 ± 1.2	5.5 ± 0.5	34.9 ± 0.8	9.8 ± 0.2	1.0 ± 0.1	3.2 ± 0.3	23.0 ± 1.0	12.2 ± 0.9	5.7 ± 0.0	1.3 ± 0.1
sucT	4.9 ± 1.0	5.7 ± 0.6	35.6 ± 0.7	9.4 ± 0.4	1.3 ± 0.8	3.2 ± 0.4	23.6 ± 0.4	10.0 ± 1.4	6.3 ± 0.9	1.3 ± 0.1
sucT comp	4.2 ± 1.0	5.4 ± 0.8	33.8 ± 1.8	8.5 ± 0.4	1.4 ± 0.5	3.2 ± 0.5	24.6 ± 1.6	12.5 ± 0.4	6.5 ± 1.5	1.1 ± 0.1
