# Supplementary material for: Role of succinyl substituents in the mannose-capping of lipoarabinomannan and control of inflammation in Mycobacterium tuberculosis infection
Source: PLoS Pathog. 2023 Sep 5;19(9):e1011636. doi: 10.1371/journal.ppat.1011636 (PMC10503756; doi:10.1371/journal.ppat.1011636)
Supplement: S6 Table — (PDF) [file ppat.1011636.s006.pdf]

S6 Table: Whole genome sequencing of WT *Mtb* CDC1551, the *sucT* transposon mutant and the complemented mutant strain, *Mtb sucT::Tn* comp-int.

Strain	Mutations	Notes
Mtb CDC1551 WT	-	Reference parent strain
Mtb sucT::Tn	Frameshift in *ponA2* (*Rv3682*): G4123558GC Frameshift in *glpK* (*Rv3696c*) A4139183AC<br><br>Both mutations were confirmed by PCR/Sanger sequencing	Inactivating frameshift mutations in *glpK* were reported to occur in clinical isolates and to accumulate in *Mtb*-infected mice (1). These mutations are rapidly reversible. They do not cause any virulence attenuation in mice but render the strains incapable of growing in medium where glycerol is the sole carbon source such as minimal Sauton's medium (2). We have confirmed that *Mtb sucT::Tn* and complemented *sucT::Tn* mutants have lost the ability to grow in Sauton's medium (data not shown).<br><br>*ponA2* disruption marginally attenuates the virulence of *Mtb* H37Rv in the lungs and spleen of C57BL/6 mice (3). This is consistent with the present study where *Mtb sucT::Tn* replicates and survives similarly to the WT parent, *Mtb* CDC1551, in macrophages.
Mtb sucT::Tn comp-int	Frameshift in *ponA2* (*Rv3682*): G4123558GC Frameshift in *glpK* (*Rv3696c*) A4139183AC<br><br>Both mutations were confirmed by PCR/Sanger sequencing	See above

References

(1) Safi H, Gopal P, Lingaraju S, Ma S, Levine C, Dartois V, et al. Phase variation in Mycobacterium tuberculosis glpK produces transiently heritable drug tolerance. Proc Natl Acad Sci U S A. 2019;116(39):19665-74. Epub 2019/09/07. doi: 10.1073/pnas.1907631116.

(2) (2) Pethe K, Sequeira PC, Agarwalla S, Rhee K, Kuhen K, Phong WY, et al. A chemical genetic screen in Mycobacterium tuberculosis identifies carbon-source-dependent growth inhibitors devoid of in vivo efficacy. Nature communications. 2010;1:57. Epub 2010/10/27. doi: 10.1038/ncomms1060.

(3) Vandal OH, Roberts JA, Odaira T, Schnappinger D, Nathan CF, Ehrt S. Acid-susceptible mutants of Mycobacterium tuberculosis share hypersusceptibility to cell wall and oxidative stress and to the host environment. J Bacteriol. 2009;191(2):625-31. Epub 2008/11/18. doi: 10.1128/JB.00932-08.
