# Supplementary material for: Role of succinyl substituents in the mannose-capping of lipoarabinomannan and control of inflammation in Mycobacterium tuberculosis infection
Source: PLoS Pathog. 2023 Sep 5;19(9):e1011636. doi: 10.1371/journal.ppat.1011636 (PMC10503756; doi:10.1371/journal.ppat.1011636)
Supplement: S7 Table — MICs were determined in 7H9-OADC-tyloxapol using the resazurin blue test and MIC values are in μg mL-1. AMP, ampicillin; CRB, carbenicillin; CIP, ciprofloxacin; RIF, rifampicin; INH, isoniazid; GEN, gentamycin; EMB, ethambutol; STR, streptomycin; CAP, capreomycin; HYG, hygromycin; IMI, imipenem. MIC determinations were performed two to three times on independent culture batches. The sucT complemented mutant strain (Mtb sucT::Tn comp) is resistant to hygromycin due to the presence of complementation plasmid, pMVGH1-Rv1565c. (PDF) [file ppat.1011636.s007.pdf]

<u>S7 Table</u>: Susceptibility of the *Mtb sucT* mutant to antibiotics.

MICs were determined in 7H9-OADC-tyloxapol using the resazurin blue test and MIC values are in µg mL^{-1}. AMP, ampicillin; CRB, carbenicillin; CIP, ciprofloxacin; RIF, rifampicin; INH, isoniazid; GEN, gentamycin; EMB, ethambutol; STR, streptomycin; CAP, capreomycin; HYG, hygromycin; IMI, imipenem. MIC determinations were performed two to three times on independent culture batches. The *sucT* complemented mutant strain (*Mtb sucT::Tn* comp) is resistant to hygromycin due to the presence of complementation plasmid, pMVGH1-*Rv1565c*.

	AMP	CRB	CIP	RIF	INH	GEN	EMB	STR	CAP	HYG	IMI
WT	> 500	625	0.31	0.02	0.32	1.90	0.80	0.32	1.25	6.20	1.90
sucT	> 500	156	0.31	0.04	0.32	1.90	0.80	0.32	1.25	6.20	0.95
sucT comp	> 500	312	0.31	0.04	0.16	1.90	0.80	0.32	1.25	> 25	1.90
