# Supplementary material for: Role of succinyl substituents in the mannose-capping of lipoarabinomannan and control of inflammation in Mycobacterium tuberculosis infection
Source: PLoS Pathog. 2023 Sep 5;19(9):e1011636. doi: 10.1371/journal.ppat.1011636 (PMC10503756; doi:10.1371/journal.ppat.1011636)
Supplement: S1 Fig — Related to Table 1. Shown are extracted ion chromatograms (EICs) of the most abundant digestion products cleaved by Cellulomonas gelida endoarabinanase from the nonreducing end of LAM purified from the different strains. Several signals with identical exact masses for Ara4 (A) and Ara6 (D) oligosaccharides with m/z values of 545.1723 [M-H]− and 809.2569 [M-H]−, respectively, reveal the possibility of more structural isomers of tetra- and hexa-arabinoside termini in Mtb LAM. Ions corresponding to Ara4+succinate at m/z 645.1887 [M-H]− (B) and Ara6+succinate at m/z 909.2732 [M-H]− (E) are missing in the sucT mutant but were detected in the LAM purified from the WT and complemented mutant strains. The most abundant mannoside-capped digestion products from Mtb WT LAM are Man2Ara4 presented as [M-H]− ions at m/z 869.2780 (C), and Man4Ara6 presented as doubly charged [M-2H]−2 ions at m/z 728.2304 [M-2H]−2 (F). These ions were not detected in the sucT mutant but their presence was restored in the complemented mutant LAM. The complemented mutant strain used in this experiment (Mtb sucT::Tn comp) expresses WT sucT from pMVGH1-Rv1565c. (PDF) [file ppat.1011636.s008.pdf]

<u>S1 Fig</u>: Negative ion liquid chromatography-mass spectrometry (LC-MS) analysis of the oligoarabinosides released by the WT, the *sucT* mutant and the complemented mutant LAM upon digestion with the *Cellulomonas gelida* endoarabinanase.

Related to Table 1. Shown are extracted ion chromatograms (EICs) of the most abundant digestion products cleaved by *Cellulomonas gelida* endoarabinanase from the nonreducing end of LAM purified from the different strains. Several signals with identical exact masses for Ara_4 **(A)** and Ara_6 **(D)** oligosaccharides with *m/z* values of 545.1723 $[M-H]^-$ and 809.2569 $[M-H]^-$, respectively, reveal the possibility of more structural isomers of tetra- and hexa-arabinoside termini in *Mtb* LAM. Ions corresponding to Ara_4+succinate at *m/z* 645.1887 $[M-H]^-$ **(B)** and Ara_6+succinate at *m/z* 909.2732 $[M-H]^-$ **(E)** are missing in the *sucT* mutant but were detected in the LAM purified from the WT and complemented mutant strains. The most abundant mannoside-capped digestion products from *Mtb* WT LAM are Man_2Ara_4 presented as $[M-H]^-$ ions at *m/z* 869.2780 **(C)**, and Man_4Ara_6 presented as doubly charged $[M-2H]^{-2}$ ions at *m/z* 728.2304 $[M-2H]^{-2}$ **(F)**. These ions were not detected in the *sucT* mutant but their presence was restored in the complemented mutant LAM. The complemented mutant strain used in this experiment (*Mtb sucT::Tn* comp) expresses WT *sucT* from pMVGH1-*Rv1565c*.

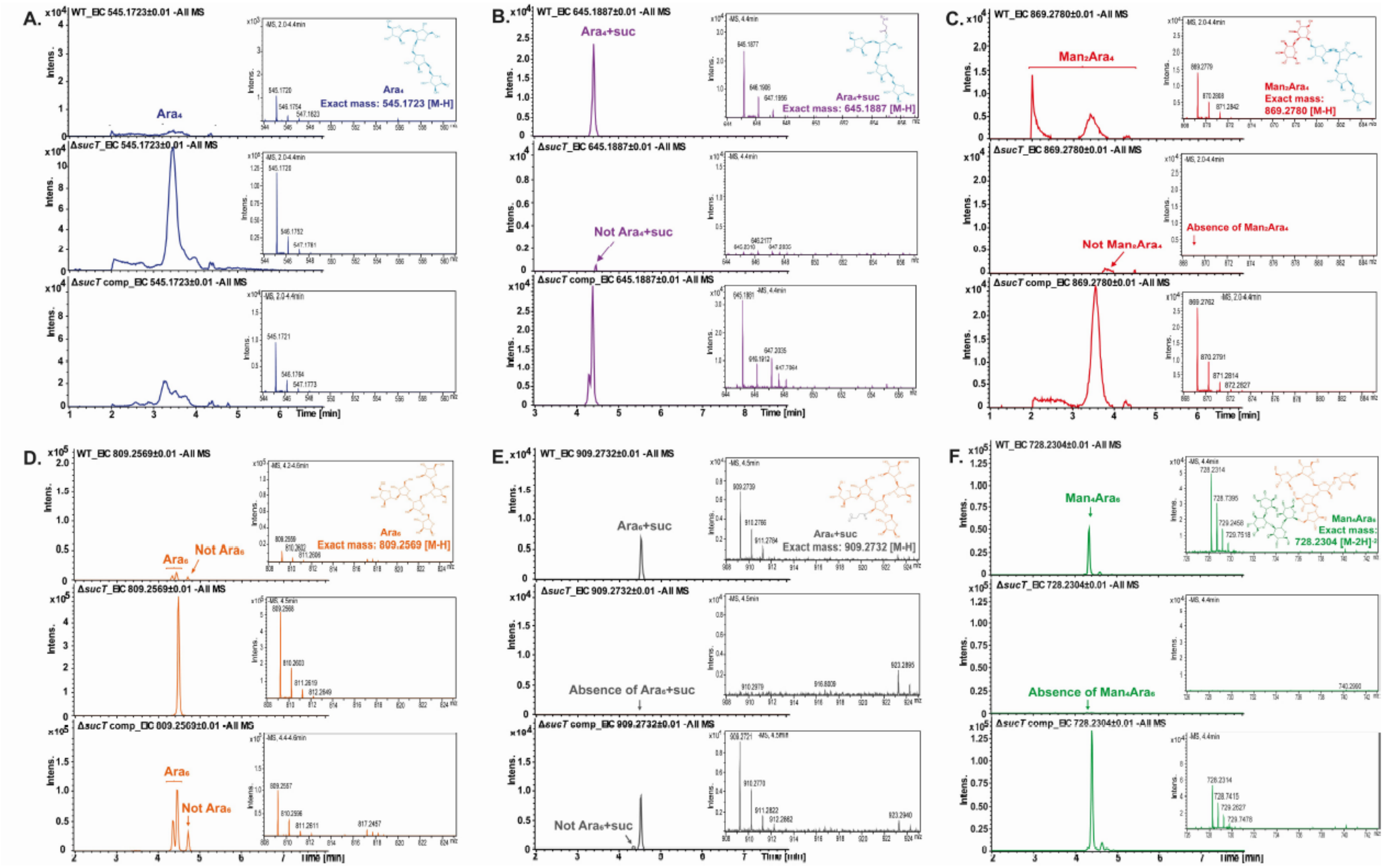

A.
WT_EIC 545.1723±0.01 -All MS
Ara₄
Exact mass: 545.1723 [M-H]
ΔsucT_EIC 545.1723±0.01 -All MS
ΔsucT comp_EIC 545.1723±0.01 -All MS
Intens.
Time [min]
B.
WT_EIC 645.1887±0.01 -All MS
Ara₄+suc
Exact mass: 645.1887 [M-H]
ΔsucT_EIC 645.1887±0.01 -All MS
Not Ara₄+suc
ΔsucT comp_EIC 645.1887±0.01 -All MS
C.
WT_EIC 869.2780±0.01 -All MS
Man₂Ara₄
Exact mass: 869.2780 [M-H]
ΔsucT_EIC 869.2780±0.01 -All MS
Absence of Man₂Ara₄
Not Man₂Ara₄
ΔsucT comp_EIC 869.2780±0.01 -All MS
D.
WT_EIC 809.2569±0.01 -All MS
Ara₆
Not Ara₆
Exact mass: 809.2569 [M-H]
ΔsucT_EIC 809.2569±0.01 -All MS
ΔsucT comp_EIC 809.2569±0.01 -All MS
E.
WT_EIC 909.2732±0.01 -All MS
Ara₆+suc
Exact mass: 909.2732 [M-H]
ΔsucT_EIC 909.2732±0.01 -All MS
Absence of Ara₆+suc
ΔsucT comp_EIC 909.2732±0.01 -All MS
Not Ara₆+suc
F.
WT_EIC 728.2304±0.01 -All MS
Man₄Ara₆
Exact mass: 728.2304 [M-2H]⁻²
ΔsucT_EIC 728.2304±0.01 -All MS
Absence of Man₄Ara₆
ΔsucT comp_EIC 728.2304±0.01 -All MS
