# Supplementary material for: Role of succinyl substituents in the mannose-capping of lipoarabinomannan and control of inflammation in Mycobacterium tuberculosis infection
Source: PLoS Pathog. 2023 Sep 5;19(9):e1011636. doi: 10.1371/journal.ppat.1011636 (PMC10503756; doi:10.1371/journal.ppat.1011636)
Supplement: S2 Fig — (A) Thin-layer chromatography analysis of total lipids extracted from WT Mtb CDC1551, the sucT mutant and the complemented mutant strain. Total lipids were loaded on aluminum-backed silica gel 60-precoated plates F254 using chloroform:methanol:water (65:25:4; by vol.) as the eluent. TLCs were revealed by spraying with cupric sulfate (i) or α-naphthol (ii) and charring. The complemented mutant strain (Mtb sucT::Tn comp) expresses WT sucT from pMVGH1-Rv1565c. (B) LC/MS analysis of PIM. Shown is the relative distribution of PIMs in WT Mtb CDC1551 (WT), the sucT mutant and the complemented mutant strain in %. PIM is used to describe the global family of phosphatidylinositol mannosides that carries one to four fatty acids (attached to the glycerol, inositol and/or mannose) and one to six mannose residues. In AcXPIMY, x refers to the number of acyl groups esterified to available hydroxyls on the mannose or myo-inositol residues, y refers to the number of mannose residues; e.g. Ac2PIM2 corresponds to the phosphatidylinositol dimannoside PIM2 carrying two acyl groups attached to the glycerol (the diacylglycerol substituent), one acyl group esterified to the mannose residue and one acyl group esterified to the myo-inositol residue. The complemented mutant strain (Mtb sucT::Tn comp) expresses WT sucT from pMVGH1-Rv1565c. (PDF) [file ppat.1011636.s009.pdf]

<u>S2 Fig</u>: PIM composition of *Mtb* CDC1551 WT, the *sucT* mutant and the complemented mutant strain.

(A) Thin-layer chromatography analysis of total lipids extracted from WT *Mtb* CDC1551, the *sucT* mutant and the complemented mutant strain. Total lipids were loaded on aluminum-backed silica gel 60-precoated plates F_{254} using chloroform:methanol:water (65:25:4; by vol.) as the eluent. TLCs were revealed by spraying with cupric sulfate (i) or α-naphthol (ii) and charring. The complemented mutant strain (*Mtb sucT::Tn* comp) expresses WT *sucT* from pMVGH1-*Rv1565c*.

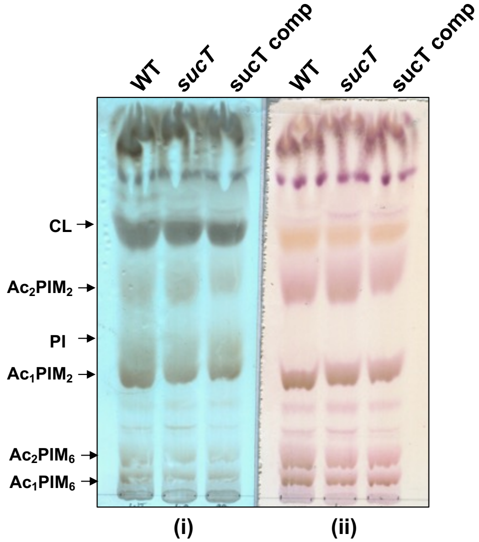

(B) LC/MS analysis of PIM. Shown is the relative distribution of PIMs in WT *Mtb* CDC1551 (WT), the *sucT* mutant and the complemented mutant strain in %. PIM is used to describe the global family of phosphatidylinositol mannosides that carries one to four fatty acids (attached to the glycerol, inositol and/or mannose) and one to six mannose residues. In Ac_XPIM_Y, x refers to the number of acyl groups esterified to available hydroxyls on the mannose or *myo*-inositol residues, y refers to the number of mannose residues; *e.g.* Ac_2PIM_2 corresponds to the phosphatidylinositol dimannoside PIM_2 carrying two acyl groups attached to the glycerol (the diacylglycerol substituent), one acyl group esterified to the mannose residue and one acyl group esterified to the *myo*-inositol residue. The complemented mutant strain (*Mtb sucT::Tn* comp) expresses WT *sucT* from pMVGH1-*Rv1565c*.

	Ac_1PIM_2 (C16,16,19)	Ac_2PIM_2 (C16,16,16,19)	Ac_2PIM_2 (C16,16,Δ18,19)	Ac_2PIM_2 (C16,16,18,19)	Ac_2PIM_2 (C16,16,19,19)	Ac_1PIM_6 (C16,16,19)	Ac_2PIM_6 (C16,16,16,19)	Ac_2PIM_6 (C16,16,Δ18,19)	Ac_2PIM_6 (C16,16,18,19)	Ac_2PIM_6 (C16,16,19,19)
WT	91.0	2.3	1.9	0.5	3.8	0.5	0.02	0.03	0.01	0.04
sucT	81.7	3.7	2.8	1.1	9.9	0.7	0.02	0.03	0.01	0.06
sucT comp	85.4	2.9	1.8	1.3	7.8	0.6	0.02	0.03	0.01	0.07
