# Supplementary material for: Role of succinyl substituents in the mannose-capping of lipoarabinomannan and control of inflammation in Mycobacterium tuberculosis infection
Source: PLoS Pathog. 2023 Sep 5;19(9):e1011636. doi: 10.1371/journal.ppat.1011636 (PMC10503756; doi:10.1371/journal.ppat.1011636)

S3 Fig: Phenotypic characterization of *Mtb sucT::Tn*.

(A) Acid-fast staining of *Mtb* CDC1551 WT, the *sucT* mutant (*sucT::Tn*) and the complemented mutant strain (*sucT::Tn* comp).

(B) Surface hydrophobicity of *Mtb* CDC1551 WT and the *sucT* mutant (*sucT::Tn*). Relative hydrophobicities were assessed by the hexadecane partition procedure as described in the Materials and Methods. The hydrophobicity index (H) is defined as the percentage reduction in the OD650 nm of the aqueous phase after partitioning with the hydrocarbon phase. The reported values are averages ± SD of three technical repeats. No statistically significant differences were noted between strains ($P > 0.05$; Student's *t*-test).

(C) Growth kinetics of WT *Mtb* CDC1551, *Mtb sucT::Tn*, *Mtb sucT::Tn comp* and *Mtb sucT::Tn comp-int* in 7H9-ADC-Tween 80 (no glycerol) at 37°C.

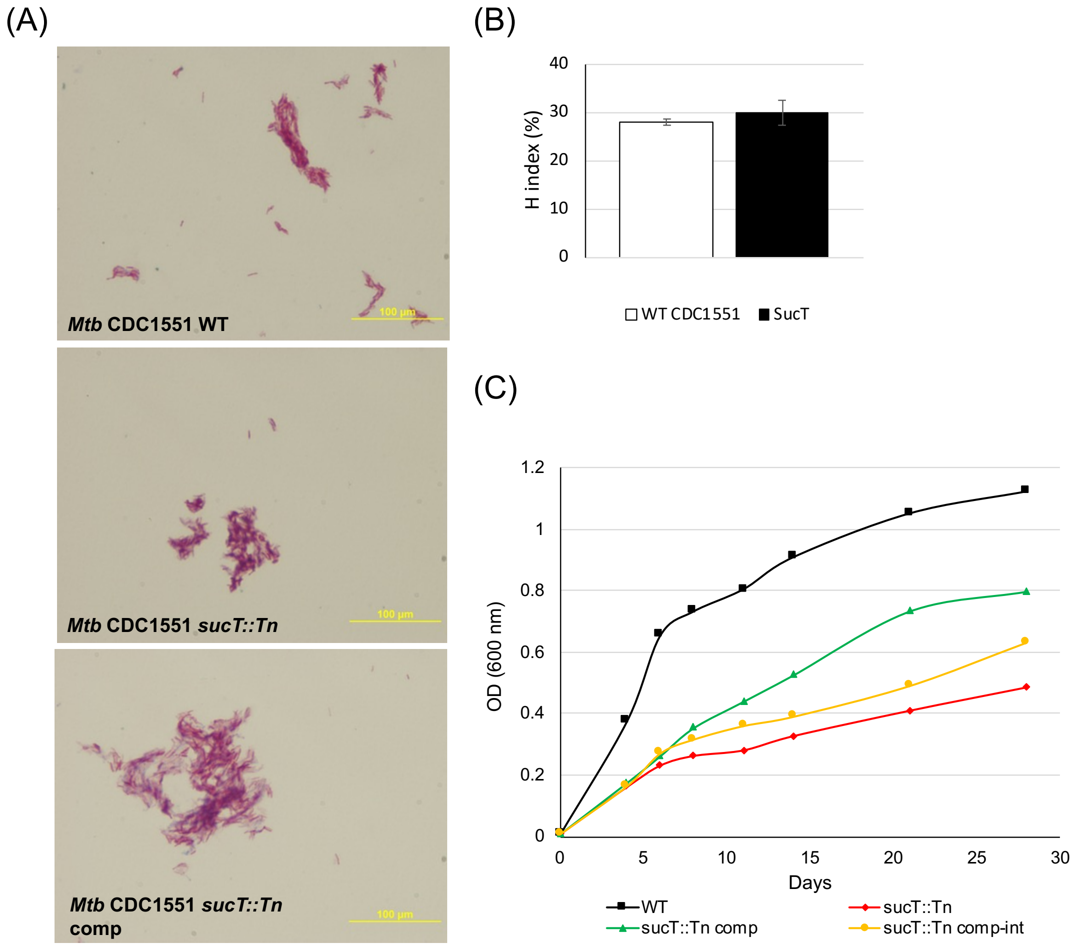

Supplement: S3 Fig — (A) Acid-fast staining of Mtb CDC1551 WT, the sucT mutant (sucT::Tn) and the complemented mutant strain (sucT::Tn comp). (B) Surface hydrophobicity of Mtb CDC1551 WT and the sucT mutant (sucT::Tn). Relative hydrophobicities were assessed by the hexadecane partition procedure as described in the Materials and Methods. The hydrophobicity index (H) is defined as the percentage reduction in the OD650 nm of the aqueous phase after partitioning with the hydrocarbon phase. The reported values are averages ± SD of three technical repeats. No statistically significant differences were noted between strains (P > 0.05; Student’s t-test). (C) Growth kinetics of WT Mtb CDC1551, Mtb sucT::Tn, Mtb sucT::Tn comp and Mtb sucT::Tn comp-int in 7H9-ADC-Tween 80 (no glycerol) at 37°C. (PDF) [file ppat.1011636.s010.pdf]
