# Supplementary material for: Role of succinyl substituents in the mannose-capping of lipoarabinomannan and control of inflammation in Mycobacterium tuberculosis infection
Source: PLoS Pathog. 2023 Sep 5;19(9):e1011636. doi: 10.1371/journal.ppat.1011636 (PMC10503756; doi:10.1371/journal.ppat.1011636)

<u>S4 Fig</u>: Comparisons of *Mtb* CFU associated with C3HeB/FeJ BMMΦ and M-CSF-differentiated human MDM 2 hours and five days post-infection.

C3HeB/FeJ BMMΦ (from Fig. 3) (A) and rM-CSF-differentiated human monocyte derived macrophages (MDM from Fig. 4) (B) were infected with either WT *Mtb* CDC1551, *Mtb sucT::Tn* or *Mtb sucT::Tn* comp and allowed to adhere for 2 h. Macrophages were subsequently lysed 2 h and 120 h post-infection and lysates were plated on 7H11-OADC agar plates for CFU counting. Shown are averages and standard deviations for triplicate wells. Data were analyzed using ordinary two-way ANOVA with * p≤0.01, **p≤0.005 and ****p≤0.0001; ns, not significant.

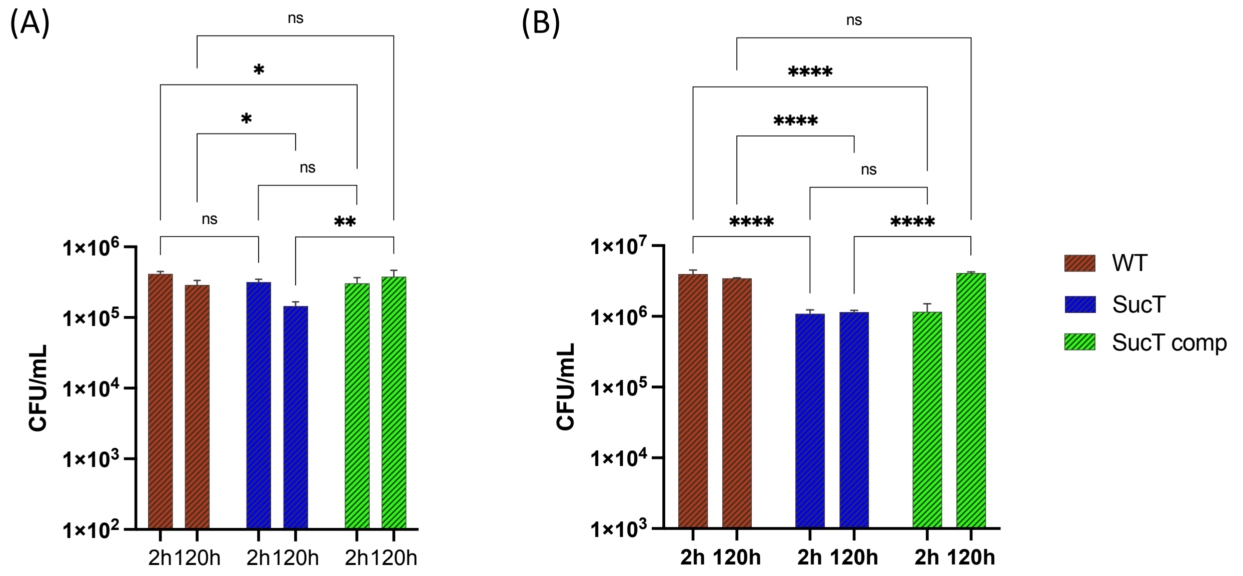

Supplement: S4 Fig — C3HeB/FeJ BMMΦ (from Fig 3) (A) and rM-CSF-differentiated human monocyte derived macrophages (MDM from Fig 4) (B) were infected with either WT Mtb CDC1551, Mtb sucT::Tn or Mtb sucT::Tn comp and allowed to adhere for 2 h. Macrophages were subsequently lysed 2 h and 120 h post-infection and lysates were plated on 7H11-OADC agar plates for CFU counting. Shown are averages and standard deviations for triplicate wells. Data were analyzed using ordinary two-way ANOVA with * p≤0.01, **p≤0.005 and ****p≤0.0001; ns, not significant. (PDF) [file ppat.1011636.s011.pdf]
